# Effect of Acute Walking on Endothelial Function and Postprandial Lipemia in South Asians and White Europeans

**DOI:** 10.1249/MSS.0000000000003098

**Published:** 2022-12-05

**Authors:** MATTHEW J. ROBERTS, ALICE E. THACKRAY, ALEX J. WADLEY, TAREQ F. ALOTAIBI, DAVID J. HUNTER, JULIE THOMPSON, KYOKO FUJIHIRA, MASASHI MIYASHITA, SARABJIT MASTANA, NICOLETTE C. BISHOP, EMMA O’DONNELL, MELANIE J. DAVIES, JAMES A. KING, THOMAS YATES, DAVID WEBB, DAVID J. STENSEL

**Affiliations:** 1 National Centre for Sport and Exercise Medicine, School of Sport, Exercise and Health Sciences, Loughborough University, Loughborough, UNITED KINGDOM; 2 National Institute for Health Research Leicester Biomedical Research Centre, University Hospitals of Leicester, National Health Service Trust and the University of Leicester, Leicester, UNITED KINGDOM; 3 School of Sport, Exercise and Rehabilitation Sciences, College of Life and Environmental Sciences, University of Birmingham, Birmingham, UNITED KINGDOM; 4 King Saud bin Abdulaziz University for Health Sciences, Respiratory Therapy Department, Riyadh, SAUDI ARABIA; 5 King Abdullah International Medical Research Centre, King Saud Bin Abdulaziz University for Health Sciences, Riyadh, SAUDI ARABIA; 6 University Hospitals of Leicester NHS Trust, Infirmary Square, Leicester, UNITED KINGDOM; 7 Graduate School of Sport Sciences, Waseda University, Tokorozawa, JAPAN; 8 Faculty of Sport Sciences, Waseda University, Tokorozawa, JAPAN; 9 Leicester Diabetes Research Centre, University of Leicester, Leicester, UNITED KINGDOM

**Keywords:** EXERCISE, FLOW-MEDIATED DILATATION, METABOLIC SYNDROME, ETHNICITY

## Abstract

**Introduction:**

South Asians (SAs) have an elevated risk of cardiovascular disease (CVD) compared with White Europeans (WEs). Postprandial endothelial function (flow-mediated dilatation (FMD%)) in SA women and SA men with central obesity has not been investigated. Research in other populations has highlighted that a 1% higher FMD% is associated with a ~13% lower risk of future CVD events. We investigated whether FMD% and lipemia, two markers for CVD risk, were higher in SAs versus WEs, whether walking improved FMD% and lipemia, and if there were ethnic differences in the response.

**Methods:**

Lean premenopausal women (study 1; 12 SA, 12 WE) and men with central obesity (study 2; 15 SA, 15 WE) completed two 2-d trials. On day 1, participants walked for 60 min at 60% of their peak oxygen uptake or rested. On day 2, participants rested and consumed two high-fat meals over 8 h. Repeated ultrasound assessments of endothelial function and venous blood samples for CVD risk markers were taken.

**Results:**

Compared with WEs, SAs had lower postprandial FMD% (study 1, −1.32%; study 2, −0.54%) and higher postprandial triacylglycerol concentrations (study 1, 0.31 mmol·L^−1^·h^−1^; study 2, 0.55 mmol·L^−1^·h^−1^). Walking improved postprandial FMD% (study 1, 1.12%; study 2, 0.94%) and resulted in no significant change or small reductions in postprandial triacylglycerol concentrations (study 1, −0.01 mmol·L^−1^·h^−1^; study 2, −0.25 mmol·L^−1^·h^−1^). Exercise-induced changes in FMD% and triacylglycerol were consistent between ethnic groups.

**Conclusions:**

Walking mitigated the adverse postprandial effect of a high-fat diet on FMD% to a similar extent in SA and WE women and men, even with no/small improvements in triacylglycerol. This study highlights the importance of exercise to clinically improve FMD% in SAs and WEs.

South Asians (SAs; people from India, Pakistan, Bangladesh, Nepal, Bhutan, Myanmar, and Sri Lanka) (1) have adverse cardiometabolic and inflammatory profiles for any given body mass index (BMI) compared with White Europeans (WEs), contributing to increased cardiovascular disease (CVD) risk (2). Consequently, SAs comprise 25% of the global population, but 60% of people with heart disease (1). In the United Kingdom, SAs suffer a 40% higher risk of coronary heart disease, a twofold to fourfold higher risk of type 2 diabetes mellitus, and experience myocardial infarctions 5 to 10 yr earlier and at a lower BMI compared with WEs (2). Accordingly, SA-specific BMI and waist circumference cut-points have been developed for the prevention of type 2 diabetes (3).

Westernized lifestyles are typified by a high intake of energy-dense foods and low physical activity, with long periods spent in the postprandial state (4). This state is characterized by increased concentrations of circulating triacylglycerol (TAG) and glucose after a meal. Over time, the repeated adverse metabolic profile seen in the postprandial state contributes to systemic inflammation and oxidative stress (5), promoting endothelial dysfunction (5) and CVD (4). Importantly, it has been proposed that SAs are genetically susceptible to the adverse metabolic profile in the postprandial state, which may coincide with postprandial lipemia-induced endothelial dysfunction that has been reported in WE populations (6). Although studies have demonstrated that healthy SA men have higher postprandial TAG concentrations than WE men (7), little is known in SA women or SA men with central obesity. Furthermore, the evidence linking postprandial TAG and endothelial function (flow-mediated dilatation; FMD%) in SAs is limited to assessments in healthy SA men in the fasted state (8).

Physical activity is important in attenuating adverse cardiovascular profiles (9). Studies from WE populations demonstrate that single bouts of moderate-to-vigorous intensity exercise improve FMD% (10) and reduce postprandial TAG concentrations in populations who are lean or living with obesity (9). Although SAs are 60% less likely to meet physical activity guidelines compared with WEs (11), it has been demonstrated that treadmill running, walking, and breaking up sitting with walking are effective at improving postprandial TAG and/or insulin concentrations in SAs (7). Importantly, the effects of exercise generally elicit greater reductions in postprandial insulin concentrations if individuals are SA compared with WE, are female compared with male, or have a BMI ≥27.2 kg·m^−2^ (12). However, assessment of metabolic markers was limited in these studies, usually to TAG, glucose, and/or insulin. Because of the lack of research in SA women who are lean and SA men with central obesity, the present studies adopted a holistic approach, investigating the effect of walking on markers of metabolic health, oxidative stress, and endothelial function.

Two studies are presented in this article. Study 1 involved SA versus WE women who were lean, whereas study 2 examined SA versus WE men with central obesity. We hypothesized that 1) SAs would exhibit lower postprandial FMD% and higher postprandial TAG concentrations than WEs, 2) walking would increase absolute postprandial FMD% and reduce postprandial TAG concentrations, and 3) walking would increase absolute FMD% and reduce postprandial TAG concentrations to a greater extent in SAs.

Secondary outcomes including anthropometric measures, magnetic resonance imaging (MRI) of fat compartments, and circulating concentrations of metabolic, inflammatory, and oxidative stress markers are reported to contribute to the understanding of primary outcomes.

## METHODS

### Ethical approval and participant recruitment

Ethical approval was granted by the institutional ethics subcommittee and registered at ClinicalTrials.gov (study 1, NCT03712501; study 2, NCT03952000). According to data from our laboratory (13), a sample size of 24 participants (12 SA and 12 WE) would have >90% power to detect a 0.41 mmol·L^−1^·h^−1^ reduction in time-averaged total area under the curve (TAUC) for TAG concentrations adopting a *P* value of 0.05.

Written informed consent was obtained from 24 premenopausal women with a lean waist circumference (<80 cm; study 1: 12 SA, 12 WE) and 30 men with central obesity (study 2: 15 SA, 15 WE) based on ethnic-specific waist circumference cut-points (≥90 cm in SA men and ≥94 cm in WE men) (3). Participants were recruited from the local community and matched between ethnic groups for age. Participants were nonsmokers, had no known CVD, and had no musculoskeletal problems (confirmed via questionnaire). Women were not taking contraceptives and participated during the early follicular phase (days 1–5) of the menstrual cycle; confirmed via plasma concentrations of 17-β estradiol and progesterone. To establish ethnicity, participants completed a verification form as reported previously (13). All WEs were North-Western Europeans (White British, French, or German) and currently living in the United Kingdom. All SA participants were currently living in the United Kingdom but had parents or grandparents born in South Asia. The verification form revealed 11 UK Indians and 1 UK Pakistani in study 1 and 11 UK Indians, 3 UK Pakistanis, and 1 Nepalese national in study 2.

### Preliminary visits

Preliminary measures included anthropometry, MRI (abdominal fat and liver proton density fat fraction), and maximal exercise tests after a short warm-up on the treadmill. In study 1, peak oxygen uptake (V̇O_2_ peak) was determined directly using an incremental test to volitional exhaustion as reported previously (14). Owing to participants’ higher CVD risk, the Bruce test was used in study 2 to predict V̇O_2_ peak (15). None of the participants reported any adverse events. Detailed methods on the preliminary measurements can be found in section 1 in the Supplemental Digital Content (Supplemental Methods, http://links.lww.com/MSS/C761).

### Main intervention

**FIGURE 1:**
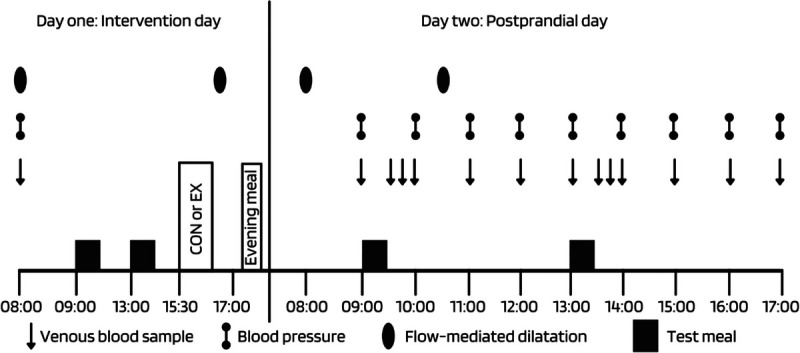
A schematic of the study design. CON, control; EX, exercise.

In both studies, participants completed two, 2-d trials in a randomized, mixed-measures, crossover design. The randomization sequence stratified by ethnicity was generated using a random number generator. In the 48 h before trials, diet was standardized while participants refrained from strenuous physical activity (≤5 min of moderate-to-vigorous intensity physical activity over 48 h confirmed via an ActiGraph GT3x activity monitor), caffeine, and alcohol. A schematic of the study design is provided in Figure 1.

On day 1 (intervention day), participants arrived at 0800 h having fasted overnight for 10 h. A resting arterial blood pressure (noninvasive; Omron M6; Omron Healthcare Co., Ltd, Kyoto, Japan), venous blood sample, and endothelial function measurement (details in “ultrasound assessment of endothelial function”) were taken. A standardized breakfast and lunch (57% fat, 32% carbohydrate, and 11% protein) were fed at 0900 and 1300 h, respectively. The breakfast provided 14.2 kcal per kilogram of body mass in both studies. The lunch provided 14.2 and 12.1 kcal per kilogram of body mass in studies 1 and 2, respectively. The energy and macronutrient content of the meals were similar to other studies investigating the effect of acute exercise on postprandial lipemia, thus facilitating comparisons between studies (13,14,16,17). The meals were well tolerated by all participants in study 1 and study 2. Details of the individual foods are presented in section 2 of the Supplemental Digital Content (Section 2: main visits, meal composition, http://links.lww.com/MSS/C761).

At 1530 h, participants rested (control) or walked (exercise) for 60 min at 60% of V̇O_2_ peak. The treadmill speed remained constant at 5.5 km·h^−1^ with the gradient adjusted to the desired exercise intensity. This was confirmed using expired air (breath-by-breath) analysis, which was also used to estimate substrate utilization and energy expenditure (18). A second endothelial function measurement was taken at ~1645 h, and participants left the laboratory at ~1700 h, after which they refrained from any strenuous physical activity and consumed a standardized pizza meal (1060 kcal, 32% fat, 52% carbohydrate, 16% protein) before 2200 h.

On day 2 (postprandial day), participants arrived at 0800 h having fasted overnight. Resting arterial blood pressure, venous blood samples, and endothelial function measurements were taken fasted and at predetermined intervals after the breakfast (0900 h) and lunch (1300 h) meals (the meals were the same as day 1; Fig. 1). Day 2 was identical for both trials with participants resting with a form of entertainment.

### Endothelial function

Endothelial function was determined via FMD% after previously published guidelines (19). The brachial artery was imaged longitudinally in the distal third of the upper arm (ML6-15 probe at 15-MHz frequency, Logic E9 ultrasound; GE Healthcare, Chicago, IL). Total shear rate was calculated using the equation 4 × mean blood velocity/internal diameter, and FMD was normalized by including shear rate as a covariate in the statistical analysis of FMD% (19). Outcome measures of interest included baseline diameter, peak diameter, flow-mediated peak diameter (FMDmm), and flow-mediated peak percentage change (FMD%). Baseline diameter was determined as the average over 60 s. Then, the blood pressure cuff was inflated to 240 mm Hg for 5 min. Further images were captured continuously for 3 min after cuff release. The scans were performed by the same researcher (M. J. R.). Images were later digitized and were analyzed offline using validated, edge-detection software (Brachial analyzer, version 4.1.3; Medical Imaging Applications, Coralville, IA) (20). Peak diameter was determined using a three-frame moving average. Detailed methods for the ultrasound assessment can be found in section 2 of the Supplemental Digital Content (Section 2: main visits, ultrasound assessments of endothelial function, http://links.lww.com/MSS/C761).

### Blood sampling

Venous blood samples were collected from an antecubital vein via venepuncture (BD Valu-Set; Becton-Dickinson, Helsingborg, Sweden) on the intervention day and cannulation (BD Ven-flon; Becton-Dickinson) on the postprandial day. All blood samples were drawn into precooled EDTA and sodium citrate monovettes (Sarstedt, Leicester, United Kingdom). Plasma total cholesterol, HDL-cholesterol, LDL-cholesterol, TAG, glucose, C-reactive protein (CRP; high sensitivity; Horiba Medical, Montpellier, France), and nonesterified fatty acid (NEFA; Randox Laboratories Ltd., County Antrim, United Kingdom) concentrations were determined spectrophotometrically using commercially available kits and a benchtop analyzer (Pentra 400; Horiba Medical, Montpellier, France). Plasma insulin (Mercodia, Uppsala, Sweden), 17-β estradiol, and progesterone (study 1 only; IBL International, Hamburg, Germany), interleukin-6 (IL-6; high sensitivity), and tumor necrosis factor-alpha (TNF-α; high sensitivity; R&D Systems, Abingdon, United Kingdom) were determined using commercially available enzyme-linked immunosorbent assays. Peroxiredoxin-4 (PRDX4) and superoxide dismutase-3 (SOD3), which are markers of oxidative stress, were quantified using in-house enzyme-linked immunosorbent assays developed using commercially available antigens and antibodies (Abcam, Cambridge, United Kingdom; Sigma Aldrich, Dorset, United Kingdom).

Detailed methods on the processing of bloods and coefficient of variation can be found in section 3 of the Supplemental Digital Content (http://links.lww.com/MSS/C761).

### Data analysis

Data were analyzed using SPSS version 23 (SPSS Inc., Chicago, IL). The model residuals were explored using histograms. Normally distributed data are presented as arithmetic means (SD), and pairwise comparisons are based on the mean differences and 95% confidence intervals (CI) of the mean absolute difference. Data that were not normally distributed were natural log transformed before analysis and are presented as geometric means (95% CI) (21). Pairwise comparisons are based on the ratio of the geometric means and 95% CI for the ratio of the geometric means (22). Absolute standardized effect sizes (Cohen’s *d*) were calculated (23).

Postprandial FMD% responses were assessed on day 2 using linear mixed models (LMM) with ethnicity (SA vs WE), trial (EX vs CON), and ethnicity–trial interaction modeled as fixed factors. Total shear rate was included as a covariate. Then, incremental FMD% (iAUC-FMD%: day 2 postprandial FMD% minus day 2 fasted FMD%) was calculated and LMM were performed with iAUC-FMD% as the outcome variable; ethnicity, trial, and ethnicity–trial interaction modeled as fixed factors; and fasted FMD% and total shear rate as covariates. This checked if results were maintained for change between fasting and postprandial values.

TAUC was calculated for TAG using the trapezoidal method for the postprandial period on day 2. Postprandial TAUC-TAG responses were assessed using LMM with ethnicity, trial, and ethnicity–trial interaction modeled as fixed factors, and fasting TAG as a covariate. Then, incremental time-averaged TAUC-TAG (iTAUC-TAG: time-averaged TAUC-TAG minus fasted TAG) was calculated and LMM were performed with ethnicity, trial, and ethnicity–trial modeled as fixed factors.

Physical characteristics and exercise responses were compared between ethnicities using LMM with ethnicity (SA vs WE) as a fixed factor. Differences in fasting values for blood pressure and plasma biomarkers were compared by averaging the fasting values on day 1 of the exercise and control trial, then performing LMM with ethnicity as a fixed factor. Time-averaged TAUC and iTAUC were calculated for blood pressure and concentrations of NEFA, glucose, insulin, PRDX4, and SOD3 using the trapezoidal method on day 2. Statistical analyses were performed for the TAUC and iTAUC responses as described for TAG. FMD% is presented in the main text, whereas baseline diameter, peak diameter, and FMDmm are presented in Supplemental Digital Content Tables 1 and 2 (Day 1 endothelial function and day 2 endothelial function, http://links.lww.com/MSS/C761). Interpretation of the data is based on the 95% CI and effect sizes rather than more conventional dichotomous hypothesis testing (24), but *P* values are presented for completeness.

## RESULTS

### Hypothesis 1: SAs exhibit lower postprandial FMD% and higher postprandial TAG concentrations than WEs

**FIGURE 2:**
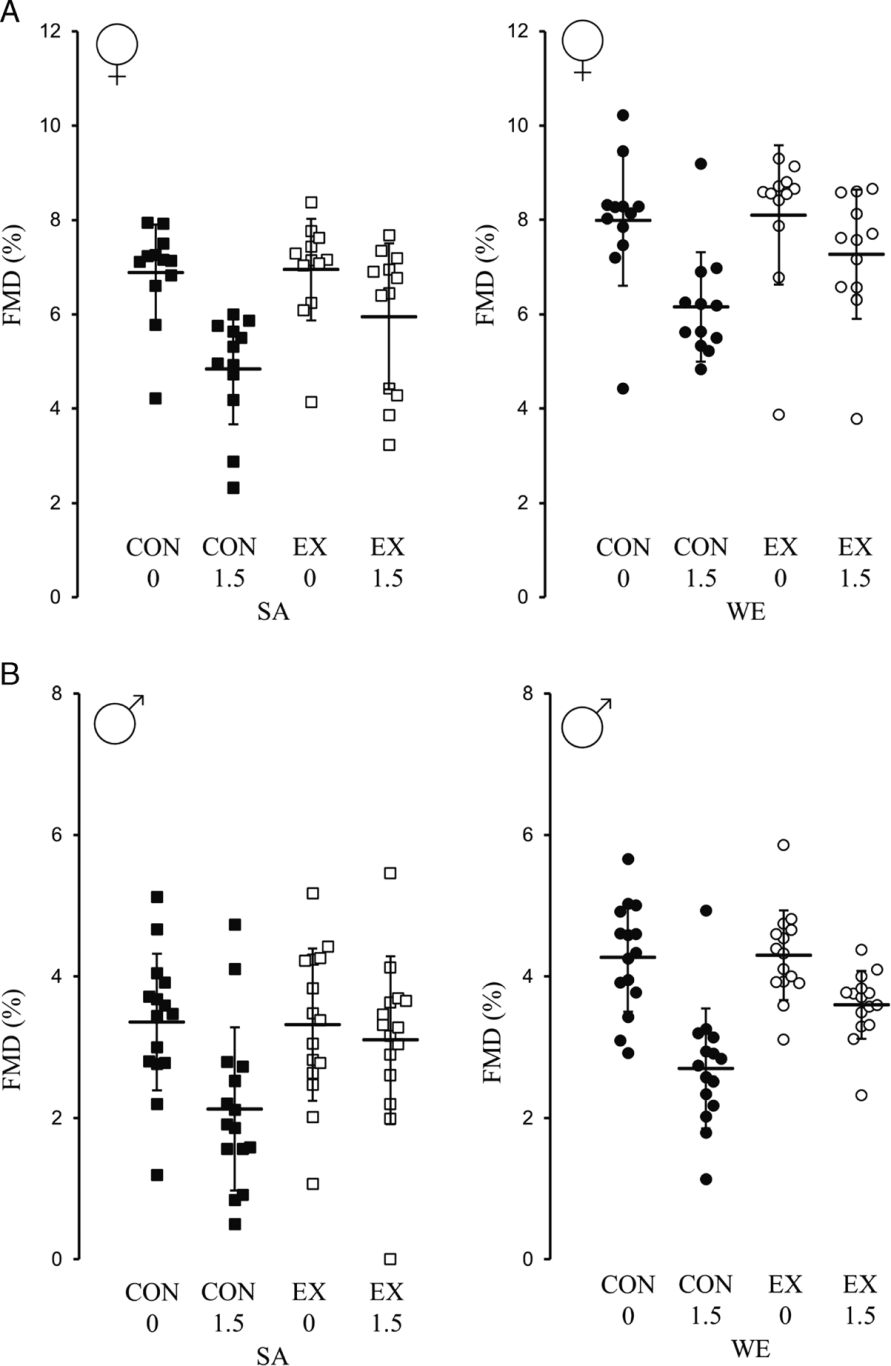
Day 2 individual FMD% changes in brachial artery diameter in the control and exercise trials for SA and WE women who were lean (A; *n* = 12 in both groups), and SA and WE men with central obesity (B; *n* = 15 in both groups). Data presented as the mean (SEM). CON, control; EX, exercise; FMD%, relative flow-mediated dilatation; 0, fasted; 1.5, 1.5 h postprandial.

A main effect of ethnicity revealed that postprandial FMD% was lower in SA versus WE women (mean difference (95% CI), −1.32% (−2.01% to −0.55%); *d* = 0.93, *P* = 0.001) and SA versus WE men (−0.54% (−1.04% to −0.04%); *d* = 0.51, *P* = 0.036; Fig. 2). After adjusting for differences in fasting FMD% between ethnicities, there was no main effect of ethnicity for iTAUC-FMD% for SA versus WE women (−0.36% (−0.90% to 0.17%); *d* = 0.18, *P* = 0.180), whereas there was a moderate effect of ethnicity for SA versus WE men (0.25% (−0.12% to 0.62%); *d* = 0.52, *P* = 0.174).

**FIGURE 3:**
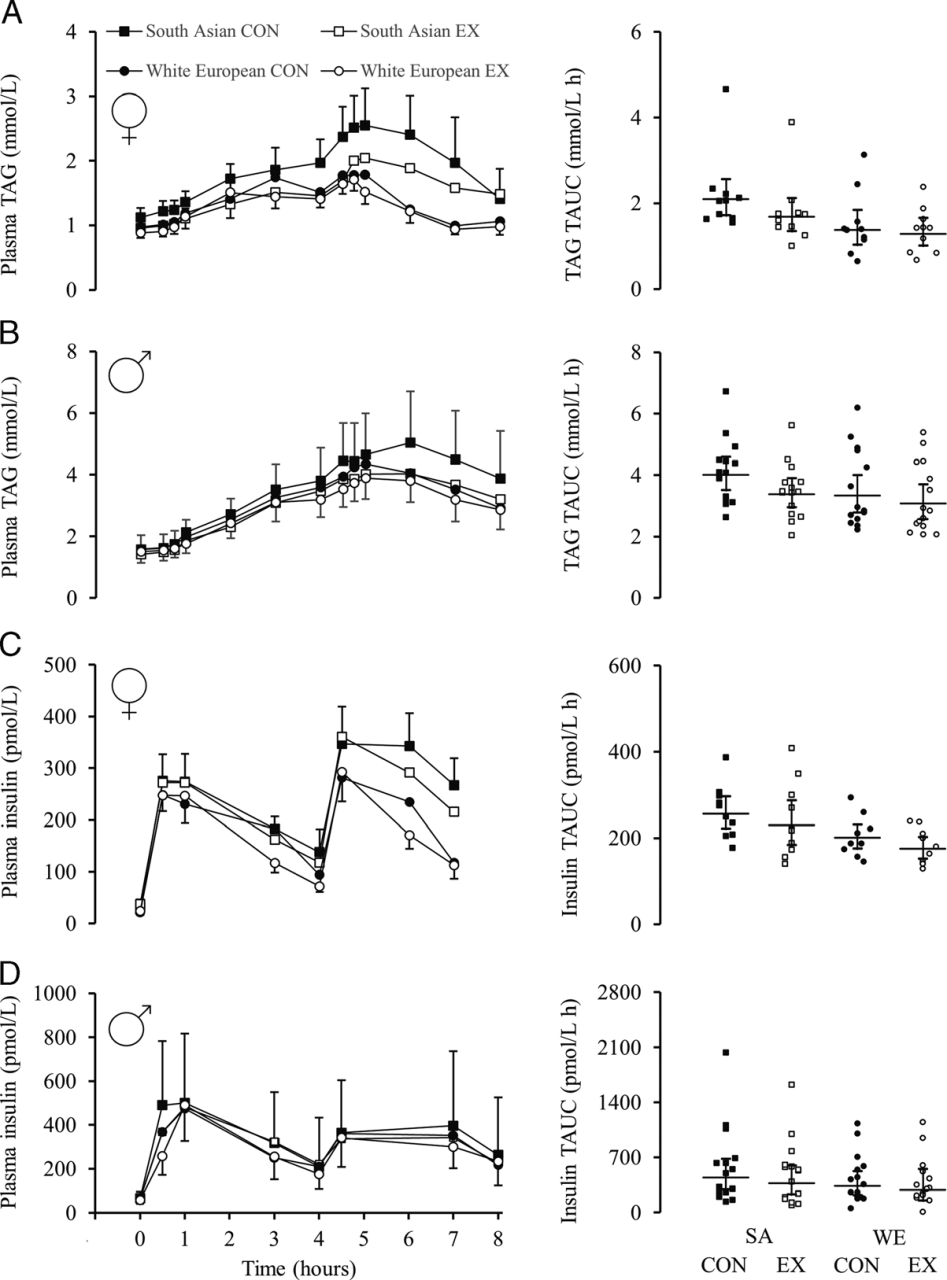
Day 2 TAG (A and B) and insulin (C and D) responses in the control and exercise trials. Panels A and C are for the women who were lean (*n* = 10 in both groups), and panels B and D are for the men with central obesity (*n* = 15 in both groups). TAG presented as the mean (SEM). Insulin presented as geometric mean (95% CI). Panels on right-side display individual time-averaged TAUC values after adjustment for differences in fasting concentrations. Breakfast fed at 0 h; lunch fed at 4 h. CON, control; EX, exercise.

After adjusting for differences in fasting TAG concentrations between ethnicities, a main effect of ethnicity revealed TAUC-TAG was higher in SA versus WE women (0.31 (0.02 to 0.61) mmol·L^−1^·h^−1^; *d* = 0.74, *P* = 0.040) and SA versus WE men (0.55 (0.20 to 0.90) mmol·L^−1^·h^−1^; *d* = 0.65, *P* = 0.003; Fig. 3, Supplemental Table 3, Supplemental Digital Content, Postprandial metabolite and blood pressure responses, http://links.lww.com/MSS/C761). A main effect of ethnicity revealed higher iTAUC-TAG for SA versus WE women (0.43 (0.01 to 0.86) mmol·L^−1^·h^−1^; *d* = 0.68, *P* = 0.045) and SA versus WE men (0.51 (0.10 to 0.92) mmol·L^−1^·h^−1^; *d* = 0.63, *P* = 0.015).

### Hypothesis 2: Walking improves absolute postprandial FMD% and TAG concentrations

A main effect of trial revealed walking improved postprandial FMD% in women (1.12% (0.35%–1.89%); *d* = 0.77, *P* = 0.006) and men (0.94% (0.44%–1.44%); *d* = 0.96, *P* < 0.001; Fig. 2). A main effect of trial also revealed that iTAUC-FMD% was higher in the exercise versus control trial for women (1.05% (0.55%–1.55%); *d* = 1.19, *P* < 0.001) and men (0.94% (0.62%–1.26%); *d* = 1.43, *P* < 0.001).

After adjusting for differences in fasting TAG concentrations between trials, walking elicited no significant reduction for TAUC-TAG in women (−0.01 (−0.31 to 0.29) mmol·L^−1^·h^−1^; *d =* 0.17, *P* = 0.968) and a small reduction for TAUC-TAG in men (−0.25 (−0.60 to 0.10) mmol·L^−1^·h^−1^; *d* = 0.39, *P* = 0.151; Fig. 3, Supplemental Table 3, Supplemental Digital Content, Postprandial metabolite and blood pressure responses, http://links.lww.com/MSS/C761). There was a small effect of walking on iTAUC-TAG for women (−0.14 (−0.56 to 0.29) mmol·L^−1^·h^−1^; *d* = 0.21, *P* = 0.508) and a moderate effect for men (−0.32 (−0.72 to 0.09) mmol·L^−1^·h^−1^; *d* = 0.65, *P* = 0.123).

### Hypothesis 3: Walking improves absolute postprandial FMD% and TAG concentrations to a greater extent in SAs than WEs

There were no ethnicity–trial interactions for postprandial FMD% in SA versus WE women (*P* = 0.998) or SA versus WE men (*P* = 0.884; Fig. 2). This finding remained for iTAUC-FMD% (*P* = 0.939 and *P* = 0.690, respectively). There were no ethnicity–trial interactions for TAUC-TAG concentrations for the women (*P* = 0.621) or men (*P* = 0.481; Fig. 3). This remained when calculating iTAUC-TAG concentrations (*P* = 0.687 and *P* = 0.489, respectively).

### Secondary outcomes

The supporting 95% CI and *P* values for secondary outcomes can be found in the relevant table(s) or figure(s) mentioned hereafter. SA women had similar percentage body fat (*d* = 0.19) but higher volumes of fat in central compartments compared with WE women (*d* ≥ 0.33), whereas SA men had lower percentage body fat (*d* = 0.37) and lower volumes of central fat (*d* ≥ 0.39) compared with WE men (Table 1). Lean mass was lower in SAs versus WEs in both studies (*d* ≥ 0.91; Table 1).

**TABLE 1:** Participant characteristics and resting blood pressure.

Variable	Women Who Were Lean				Men with Central Obesity			
	SA (*n* = 12)	WE (*n* = 12)	Pairwise Comparisons SA vs WE Women		SA (*n* = 15)	WE (*n* = 15)	Pairwise Comparisons SA vs WE Men	
			Mean Difference (95% CI)	Effect Size (*d*)			Mean Difference (95% CI)	Effect Size (*d*)
Age (yr)	24.2 (6.2)	24.3 (5.4)	−0.2 (−5.1 to 4.8)	0.03	41.3 (11.3)	41.6 (12.7)	−0.3 (−9.3 to 8.7)	0.03
Stature (cm)	163.9 (4.8)	166.0 (6.1)	−2.1 (−6.7 to 2.5)	0.38	172.7 (6.2)	181.5 (3.3)	−8.8(−12.5 to −5.0)*	1.76
Body mass (kg)	62.5 (10.7)	65.4 (8.5)	−2.9 (−11.1 to 5.3)	0.30	93.3 (17.2)	112.8 (18.5)	−19.5(−32.9 to − 6.2)*	1.09
BMI (kg·m^−2^)	23.3 (3.8)	23.9 (4.0)	−0.6 (−3.9 to 2.7)	0.16	31.5 (7.2)	34.3 (5.5)	−2.7 (−7.6 to 2.1)	0.42
Lean mass (kg)	42.5 (4.4)	47.0 (5.5)	−4.5(−8.8 to −0.3)*	0.91	61.2 (6.4)	71.2 (6.8)	−9.9(−14.9 to −5.0)*	1.50
Body fat (%)*^a^*	31.1 (7.3)	29.9 (5.8)	1.2 (−4.3 to 6.8)	0.19	33.3 (7.4)	36.0 (7.4)	−2.7 (−8.3 to 2.8)	0.37
Waist circumference (cm)	76.3 (3.1)	77.3 (2.5)	−0.9 (−2.8 to 1.9)	0.07	106.3 (15.0)	113.7 (14.0)	−7.4 (−18.3 to 3.5)	0.51
Total abdominal fat (L)*^b^*	6.79 (3.86)	5.70 (1.89)	1.09 (−1.76 to 3.95)	0.36	14.61 (6.76)	18.72 (6.93)	−4.11 (−9.33 to 1.11)	0.60
Visceral adipose tissue (L)*^b^*	1.14 (0.93)	0.93 (0.27)	0.21 (−0.39 to 0.82)	0.33	4.21 (1.74)	6.19 (2.05)	−1.99(−3.43 to −0.54)*	1.05
Abdominal subcutaneous adipose tissue (L)*^b^*	5.64 (3.01)	4.77 (1.67)	0.88 (−1.41 to 3.16)	0.36	10.41 (5.24)	12.53 (5.61)	−2.12 (−6.25 to 2.01)	0.39
Visceral adipose tissue ratio (%)*^c^*	15.6 (4.5)	16.8 (3.5)	−1.2 (−5.0 to 2.5)	0.31	29.6 (6.8)	34.2 (8.5)	−4.6 (−10.4 to 1.3)	0.59
Visceral adipose tissue index (L·m^−2^)*^d^*	0.43 (0.33)	0.35 (0.11)	0.08 (−0.14 to 0.32)	0.35	1.42 (0.58)	1.88 (0.61)	−0.46(−0.91 to −0.01)*	0.77
Fat index (L·m^−2^)^e^	2.58 (1.46)	2.12 (0.72)	0.46 (−0.62 to 1.55)	0.40	4.94 (2.25)	5.68 (2.04)	−0.74 (−2.39 to 0.90)	0.35
Liver fat fraction (%)*^b^*	1.66 (1.61)	1.61 (1.07)	0.04 (−1.24 to 1.32)	0.03	9.21 (8.42)	12.21 (9.03)	−3.00 (−9.60 to 3.60)	0.34
Peak oxygen uptake (L·min^−1^)	2.16 (0.35)	2.62 (0.49)	−0.46(−0.82 to −0.10)*	1.09	2.66 (0.66)	3.55 (0.75)	−0.88(−1.41 to −0.36)*	1.26
Peak oxygen uptake (mL·kg^−1^·min^−1^)	34.8 (5.1)	40.5 (8.5)	−5.7 (−11.6 to 0.3)	0.81	29.2 (7.7)	31.7 (5.2)	−2.5 (−7.4 to 2.4)	0.38
Resting SBP (mm Hg)	109 (104 to 115)	115 (109 to 121)	−5% (−11% to 2%)	0.03	122 (13)	132 (14)	−10(−18 to −1)*	0.79
Resting DBP (mm Hg)	72 (69 to 76)	71 (68 to 75)	1% (−6% to 9%)	0.01	76 (10)	81 (9)	−5 (−11 to 1)	0.58

Within groups, normally distributed data are mean (SD) and nonnormally distributed data are geometric mean (95% CI). For normally distributed data, ethnic comparisons are based on the mean absolute difference (95% CI of the mean absolute difference between ethnicities). For nonnormally distributed data, ethnic comparisons are based on the ratio of the geometric means (95% CI for the ratio of geometric means between ethnicities).

Significant differences are highlighted using bold text.

*^a^*Body fat determined by bioelectrical impedance.

*^b^*Total abdominal fat, visceral adipose tissue, abdominal subcutaneous adipose tissue, and liver fat fraction determined via MRI.

*^c^*Visceral adipose tissue ratio determined via MRI. This is visceral fat divided by the total abdominal fat.

*^d^*Visceral adipose tissue index determined via MRI. This is the total amount of visceral fat divided by height squared.

*^e^*Fat index determined via MRI. This is the total amount of abdominal fat divided by height squared.

*Main effect of ethnicity (*P* ≤ 0.047).

DBP indicates diastolic blood pressure; SBP, systolic blood pressure.

In both studies, SAs generally had higher fasting concentrations of circulating inflammatory markers (IL-6, CRP, TNF-α; *d* ≥ 0.38 apart from CRP in women *d* = 0.08), higher fasting LDL-cholesterol concentrations (*d* ≥ 0.54), and lower V̇O_2_ peak (*d* ≥ 0.38) compared with WEs (Supplemental Table 4, Supplemental Digital Content, Fasting plasma metabolite concentrations and baseline relative flow-mediated dilatation, http://links.lww.com/MSS/C761). SA women had lower fasting HDL-cholesterol concentrations than WE women (*d* = 1.07), but this was not seen in men (*d* = 0.06; Supplemental Table 4, Supplemental Digital Content, http://links.lww.com/MSS/C761). SA men had lower resting arterial systolic blood pressure and diastolic blood pressure than WE men (*d* ≥ 0.58; Supplemental Table 4, Supplemental Digital Content, http://links.lww.com/MSS/C761).

SAs had lower absolute and relative peak oxygen uptake (*d* ≥ 0.66), oxidized less carbohydrate (*d* ≥ 0.67), and had lower total energy expenditure during exercise compared with WEs (*d* ≥ 1.09; Supplemental Table 5, Supplemental Digital Content, Exercise responses, http://links.lww.com/MSS/C761). Furthermore, SA men oxidized less fat during exercise than WEs (*d* = 0.64), but this was not seen in women (*d* = 0.03). Postprandial TAUC responses for blood pressure, insulin, glucose, NEFA, PRDX4, and SOD3 can be found in the Supplemental Digital Content (Supplemental Results, http://links.lww.com/MSS/C761).

## DISCUSSION

The main findings of the present studies are the following: 1) postprandial FMD% was lower in SA versus WE women who were lean, and SA versus WE men with central obesity; 2) postprandial TAUC-TAG concentrations were higher in SAs versus WEs; 2) walking improved postprandial FMD% in SAs and WEs and did not improve TAUC-TAG concentrations in SA and WE women, but elicited a small reduction for SA and WE men; and 3) there were no ethnicity–trial interactions for postprandial FMD% or TAG concentrations.

Fasting and postprandial FMD% were 1.21% and 1.32% lower in SA versus WE women, and 1.01% and 0.54% lower in SA versus WE men, respectively. This holds clinical importance as a 1% higher FMD% is associated with a ~13% lower risk of future CVD events (25). The lower postprandial FMD% in SAs was accompanied by higher postprandial TAG concentrations. Higher postprandial TAG concentrations in SAs may be due to lower muscle lipoprotein lipase activity, impairing the removal of TAG from the circulation (26). Studies in SAs have highlighted that high insulin concentrations and/or insulin resistance may exaggerate the postprandial TAG response by inhibiting the normal suppressive action of insulin on hepatic very-low-density-lipoprotein production (7). Furthermore, insulin resistance and hyperinsulinemia promote CVD by increasing vascular stiffness (27). However, insulin concentrations are unlikely to explain the current findings as SA men had similar concentrations to WE men, and there was little effect of exercise on the insulin concentrations in men. Instead, the higher postprandial TAG concentrations in SAs versus WEs may contribute to the lower postprandial FMD% as postprandial hypertriglyceridemia can induce endothelial dysfunction via enhanced oxidative stress. For example, long-term simvastatin treatment (a lipid-lowering drug) has been shown to lower postprandial hypertriglyceridemia and oxidative stress, improving endothelial function (28).

No research so far has investigated the influence of inflammatory and oxidative stress markers in SAs, which may modulate the lower FMD% in the fasted state. In the present studies, the lower FMD% in SAs across the fasted and postprandial state may be associated with adverse inflammatory profiles. Circulating inflammatory profiles (CRP, TNF-α, and IL-6) were generally higher in SAs than WEs, and this may provoke endothelial damage (29), increases in reactive oxygen species, and disturbances in nitric oxide (NO) bioavailability, thus reducing the vasodilatory effect of NO (30). Consequently, IL-6 and TNF-α are associated with heart failure and all-cause mortality (31). Therefore, it is possible that the lower FMD% in SAs was a consequence of unfavorable inflammatory profiles contributing to vascular dysfunction by disrupting the homeostatic balance of vasoactive factors within the vasculature (5).

The relationship between inflammation and vascular dysfunction involves oxidative stress. It was expected that both PRDX4 and SOD3 (markers of oxidative stress) would be higher in SA versus WEs, but PRDX4 was lower and SOD3 was higher in SA versus WE women, whereas SOD3 was lower and PRDX4 was similar in SA versus WE men. High concentrations of PRDX4 are found in the blood of patients with sepsis and may reflect an antioxidant system in imbalance, like those with atherosclerosis (32). The present findings are surprising and warrant further investigation. Limited evidence in lean SA men has also found a low number and functionality of endothelial progenitor cells due to low NO bioavailability, and this may contribute to the lower FMD% in SAs in the present studies (33). Future studies should investigate the complex relationship between ethnicity, inflammation, NO bioavailability, endothelial progenitor cell mobilization, and endothelial function. This may be particularly important, as it seems that ethnic differences in fat storage do not explain the ethnic variation in CVD risk (34).

Another key finding was that postprandial FMD% improved the day after exercise compared with the control trial, even after adjusting for fasting FMD%. After exercise, there is a biphasic response in endothelial function that varies depending on exercise type, endothelial stimuli (baseline diameter, oxidative stress, shear stress, and NO bioavailability), and participant characteristics (cardiorespiratory fitness) (10). Endothelial function decreases or stays the same immediately after exercise, but increases ~1–24 h after exercise and remains increased for up to ~48 h after exercise in healthy populations (10). This was seen in the present studies in both ethnicities, with walking improving postprandial FMD%, even after adjustment for differences in fasting FMD% between trials. Again, the risk of future CVD events is 13% higher per 1% lower FMD% (25), so these findings highlight the importance of walking to clinically improve endothelial function.

It is well documented that exercise reduces postprandial TAG concentrations for up to ~20 h after exercise (9). Research in healthy SA versus WE men who were lean has demonstrated that 60 min of running at 70% of V̇O_2_ max decreased postprandial TAG concentrations to a greater extent in SAs versus WEs (22% vs 10%, respectively) (16), whereas 60 min of walking at 60% of V̇O_2_ max decreased postprandial TAG concentrations to a similar extent in SAs versus WEs (8% vs 10%, respectively) (13). Therefore, greater exercise intensities and/or energy expenditure may be needed to maximize reductions in postprandial TAG concentrations in SAs (9). In the present studies, a 60-min walk at 60% of V̇O_2_ peak resulted in no significant reduction in TAUC-TAG concentrations for women and a small reduction in TAUC-TAG concentrations for men, regardless of ethnicity.

The small improvement in postprandial TAG concentrations during the exercise trial in men may contribute to the increased FMD%. Previous studies in physically active and sedentary adults have demonstrated a close link between higher postprandial TAG concentrations and lower endothelial function, partly due to hypertriglyceridemia inducing oxidative stress, impairing endothelial function (28). This could be problematic as many people living in Westernized countries consume energy-dense, high-fat meals and snacks throughout the day, experiencing a near continuous cycle of elevated TAG concentrations and endothelial dysfunction, contributing to the development of CVD (6). Therefore, the results of the present studies suggest that walking may be effective at attenuating the cycle of elevated TAG concentrations and endothelial dysfunction in men, but the mechanisms need further investigation—particularly in women—with a focus on oxidative stress and inflammation. If an exercise-induced improvement of TAG regulation and endothelial dysfunction was caused by an improvement in oxidative stress, then both PRDX4 and SOD3 would have decreased in the exercise versus control trial for both studies. This was only seen for the women, as SOD3 was unchanged in the exercise versus control trial for men.

Given the high levels of physical inactivity in SAs (11) and the relationship between postprandial TAG concentrations, endothelial dysfunction, type 2 diabetes, and CVD risk (35), the current studies offer promising findings on the beneficial effects of moderate-intensity walking in SA people and highlight key areas for further research. Although this study had several strengths including well-controlled conditions and precise assessment techniques such as ultrasound and MRI, it is important to note some limitations. Although walking may be a feasible intensity to exercise, higher exercise intensities should be examined as they are likely to elicit greater responses. SAs are a heterogeneous population, and most of the participants identified as Indian SAs. These findings require confirmation in other SA groups (e.g., Bangladeshis and Pakistanis). Polycystic ovary syndrome is more prevalent in SA women and is related to higher insulin concentrations (36). Although menstrual hormones were similar in the current study, insulin resistance is central to the pathogenesis of polycystic ovary syndrome and may be a confounding factor for research in SA women. Endothelium-independent function was not investigated, and inclusion may provide novel information about structural changes of the vessel wall. Circulating NO levels were not measured, and this may have identified possible mechanisms of ethnic differences in FMD%.

## CONCLUSIONS

In conclusion, these studies observed that fasting and postprandial endothelial function were lower in SA versus WE women who were lean and SA versus WE men with central obesity. Furthermore, postprandial TAUC-TAG concentrations were higher in SA versus WEs, but moderate-intensity walking, which was well tolerated by participants, improved postprandial endothelial function even with small-to-no significant reductions in TAG the day after exercise. These findings indicate that walking is effective at attenuating vascular changes occurring after ingesting dietary fat, even with small metabolic changes. Thus, the studies contribute to our understanding of the benefits of exercise in reducing CVD risk in SAs.

## Supplementary Material

SUPPLEMENTARY MATERIAL
