# Supplementary material for: Effect of Acute Walking on Endothelial Function and Postprandial Lipemia in South Asians and White Europeans
Source: Med Sci Sports Exerc. 2022 Dec 5;55(5):794–802. doi: 10.1249/MSS.0000000000003098 (PMC10090289; doi:10.1249/MSS.0000000000003098)
Supplement: SUPPLEMENTARY MATERIAL [file msse-55-794-s001.docx]

**Effect of acute walking on endothelial function and postprandial lipaemia in South Asians and white Europeans**

Matthew J. Roberts, Alice E. Thackray, Alex J. Wadley, Tareq F. Alotaibi, David J. Hunter, Julie Thompson, Kyoko Fujihara, Masashi Miyashita, Sarabjit Mastana, Nicolette C. Bishop, Emma O’Donnell, Melanie J. Davies, James A. King, Thomas Yates, David Webb, David J. Stensel

**Supplementary information.**

The order of the supplementary information coincides with appearance in the main text.

**Supplemental methods:** page 2.

**Supplemental results:** page 5.

**Supplemental tables:** page 7.

**Supplemental figures:** page 32.

**Supplemental methods**

***Section one: preliminary measures.***

Height and body mass were measured using an integrated stadiometer and digital scale (Seca Ltd, Hamburg, Germany), and BMI was subsequently calculated. Body fat percentage was measured using bioelectrical impedance analysis in a fasted state, one hour after the consumption of 250 mL of water (Seca Ltd, Hamburg, Germany). Waist circumference was measured using a flexible plastic tape midway between the lower costal margin and the iliac crest after normal expiration (Hokanson, Washington, USA).

Treadmill exercise tests were undertaken on a motorised treadmill (ExciteMed, Technogym, Cesena, Italy). Heart rate (Polar T31, Polar Electro, Kempele, Finland) and expired air (Metalyser 3B, Cortex, Leipzig, German) were measured continuously throughout the tests, and perceived exertion was measured at the end of each stage during sub-maximal tests. In study one, V̇O_2_ max was determined directly using an incremental test to volitional exhaustion as reported previously^14^. Owing to their higher CVD risk, the Bruce test was used in study two to predict V̇O_2_ peak.

Magnetic resonance imaging (MRI; GE 3.0T Discovery MR750w, General Electric, Boston, USA) was used to quantify abdominal fat and liver proton density fat fraction. A T1-weighted 3D FSPGR LAVA-flex sequence with neck-to-knee coverage was used to determine total abdominal fat, abdominal subcutaneous adipose tissue, VAT, a VAT index (absolute visceral fat divided by height squared), and an abdominal fat index (absolute abdominal fat divided by height squared). The IDEAL-IQ sequence was used to assess proton density fat fraction in the liver. Anonymised scans were later analysed by AMRA Medical using their AMRA^TM^ profiler (AMRA Medical AB, Linkoping, Sweden).

***Further details on the MRI outcomes and how they were assessed.***

- Total abdominal fat (L) – Abdominal subcutaneous adipose tissue plus visceral adipose tissue.
- Visceral adipose tissue (L) – Adipose tissue within the abdominal cavity, excluding adipose tissue outside the abdominal skeletal muscles, and adipose tissue and lipids within and posterior of the spine and posterior of the back muscles.
- Abdominal subcutaneous adipose tissue (L) – Subcutaneous adipose tissue in the abdomen from the top of the femoral head to the top of the thoracic vertebrae T9.
- Visceral adipose tissue ratio (%) – Visceral fat divided by the total abdominal fat.
- Visceral adipose tissue index (L/m^2^) – Total amount of visceral fat divided by height squared. This is like BMI, but only sensitive for visceral fat.
- Fat index (L/m^2^) – Total amount of abdominal fat divided by height squared. This is like BMI, but only sensitive for truncal fat.
- Liver fat fraction (%) – measured as the average proton-density fat fraction in manually placed regions of interest, avoiding major vessels and bile ducts.

***Section two: main visits.***

***Meal composition:***

The breakfast consisted of plain croissants, chocolate spread, chocolate milkshake, and double cream which provided 14.2 kcal per kilogram of body mass (57% fat, 32% carbohydrate, 11% protein). The lunch consisted of white bread, Cheddar cheese, butter, and chocolate milkshake which provided 14.2 kcal and 12.1 kcal per kilogram of body mass in study one (women) and study two (men), respectively (57% fat, 32% carbohydrate, 11% protein). Water was available ad libitum.

***Ultrasound assessments of endothelial function:***

Endothelial function was determined via flow-mediated dilatation (FMD) using previously published guidelines and recommendations^17^. Briefly, a pneumatic blood pressure cuff (Hokanson, Washington, USA) was placed on the right arm immediately distal to the elbow. Participants rested in a supine position within a temperature controlled and darkened room for 20 minutes, with their right arm extended approximately 90° from their torso. The brachial artery was imaged longitudinally in the distal third of the upper arm (ML6-15 probe at 15 MHz frequency, Logic E9 ultrasound, GE healthcare, Chicago, USA). A baseline scan was conducted over 60 seconds. Then, the blood pressure cuff was inflated to 240 mmHg for five minutes. Further images were captured continuously after cuff release for three minutes. Images were captured on the r-wave of each cardiac cycle (Vascular Imager, Version 4.1.3, Medical Imaging Applications, Iowa, USA). Ultrasound settings (contrast, focus, and depth) were modified to optimise images of the arterial wall interface and used for each participants’ subsequent scans. Participant specific landmarks, ink markers, measures of distance from the elbow and shoulder, and photographs were used to maintain consistency in scan location. Pulsed wave doppler was used simultaneously with beta-mode imaging to assess blood flow velocity at an isonation angle ≤60°. The scans were performed by the same researcher who had received six months of training and had <2% coefficient of variance scores in over 100 individuals. Recorded images were later digitised and analysed using specialised, validated edge-detection software (Brachial Analyser, Version 4.1.3, Medical Imaging Applications, Iowa, USA). Peak diameter was determined using a three-frame moving average. Flow-mediated dilatation was expressed in absolute terms (FMDmm) and as the percentage change from basal diameter to peak diameter (FMD%).

***Section three: processing of bloods and coefficient of variation***

EDTA monovettes were spun immediately in a refrigerated centrifuge at 3500 x g for 10 minutes at 4°C (Labofuge 400R, ThermoScientific, Langenselbold, Germany). Sodium citrate monovettes were immediately centrifuged at 2500 x g for 15 minutes at 19°C (Labofuge 400R, ThermoScientific, Langenselbold, Germany). Plasma was subsequently aliquoted and stored at -80°C before analysis.

The within-batch coefficient of variation for each assay was as follows: 1.2% for TAG, 0.8% TC, 0.7% for HDL, 0.8% for LDL, 1.1% for NEFA, 0.7% for glucose, 2.9% for insulin, 0.8% for CRP, 3.9% for IL-6, 4.4% for TNF-α, 5.1% for PRDX4, and 4.9% for SOD3.

**Supplemental results**

***Secondary outcomes – postprandial metabolite concentrations and blood pressure responses.***

On day two, SA women had higher TAUC for NEFA and SOD3 with a moderate-large effect size (*d*≥0.51), and higher concentrations of insulin with a small effect size compared with WE women (*d*=0.26) (Figure 3, Supplementary Figures 1 and 2, Supplementary Table 3). SA women also had lower TAUC for SBP and PRDX4 with a moderate-large effect size (*d*≥0.57) (Supplementary Figure 2 and 3, Supplementary Table 3). SA men had higher DBP (*d*=0.50), but lower glucose, NEFA, SOD3 and SBP (*d*≥0.23) than WE men (Supplementary Figures 1-3, Supplementary Table 3).

On day two, TAUC for insulin, NEFA, PRDX4 and SOD3 were lower in the exercise vs. control trial for women (*d*≥0.23) (Figure 3, Supplementary Figures 1 and 2, Supplementary Table 3). TAUC for NEFA increased in the exercise vs. control trial for men (*d*=0.86), but PRDX4, SBP and DBP all decreased (*d*≥0.22) (Supplementary Figures 1,2.3, Supplementary Table 3).

Ethnicity-by-trial interactions revealed an effect for NEFA and PRDX4 TAUC (P≤0.047) in the women who were lean. NEFA TAUC increased in the exercise vs. control trial for SA women (8 (-11 to 31)%) and decreased in the exercise vs. control trial for WE women (-16 ( -26 to -6)%). PRDX4 TAUC decreased to a lesser extent in the exercise vs. control trial for SA women (-0.06 (-1.38 to 1.26) ng/mL h) than WE women (-1.94 (-3.37 to -0.52) ng/mL h). An ethnicity-by-trial interaction revealed an effect for DBP TAUC in the men with central obesity. DBP TAUC decreased in the exercise vs. control trial for SA men (-3 (-4 to -1)mmHg h) but not in WE men (0 (-2 to 3) mmHg h).

**Supplementary Table 1. Day 1 endothelial function.** The baseline diameter of the brachial artery, peak diameter, absolute FMD (FMDmm), and relative FMD (FMD%) across day 1 during the control and exercise trial for South Asian and white European women who were lean, and South Asian and white European men with central obesity.

| **Variable** | **SA women who were lean**  **(*n* = 12)** | | | | **WE women who were lean**  **(*n* = 12)** | | | | **Pairwise comparisons SA vs. WE** | | **Pairwise comparison EX vs. CON** | |
| --- | --- | --- | --- | --- | --- | --- | --- | --- | --- | --- | --- | --- |
|  | **CON**  **(fasted)** | **EX**  **(fasted)** | **CON**  **(PP)** | **EX**  **(PP)** | **CON**  **(fasted)** | **EX**  **(fasted)** | **CON**  **(PP)** | **EX**  **(PP)** | **Mean difference (95% CI)** | **Effect size (*d*)** | **Mean difference (95% CI)** | **Effect size (*d*)** |
| Baseline diameter (mm) | 3.69  (0.65) | 3.68  (0.64) | 3.69  (0.63) | 3.73  (0.62) | 3.52  (0.54) | 3.51  (0.56) | 3.50  (0.55) | 3.52  (0.56) | 0.19  (-0.32 to 0.69) | 0.31 | 0.01  (-0.01 to 0.03) | 0.02 |
| Peak diameter (mm) | 3.94  (0.69) | 3.94  (0.69) | 3.90  (0.67) | 3.92  (0.66) | 3.80  (0.58) | 3.79  (0.60) | 3.74  (0.59) | 3.76  (0.58) | 0.15  (-0.38 to 0.69) | 0.24 | 0.01  (-0.01 to 0.03) | 0.01 |
| **Variable** | **SA women who were lean**  **(*n* = 12)** | | | | **WE women who were lean**  **(*n* = 12)** | | | | **Pairwise comparisons SA vs. WE** | | **Pairwise comparison EX vs. CON** | |
|  | **CON**  **(fasted)** | **EX**  **(fasted)** | **CON**  **(PP)** | **EX**  **(PP)** | **CON**  **(fasted)** | **EX**  **(fasted)** | **CON**  **(PP)** | **EX**  **(PP)** | **Mean difference (95% CI)** | **Effect size (*d*)** | **Mean difference (95% CI)** | **Effect size (*d*)** |
| Flow mediated dilatation (%) | 6.93  (1.19) | 6.94  (1.17) | 5.58  (1.02) | 5.26  (1.70) | 8.14  (1.36) | 8.02  (1.23) | 6.85  (0.84) | 6.80  (0.86) | **-1.27**  **(-2.15 to -0.39)*** | 1.08 | -0.12  (-0.40 to 0.16) | 0.10 |
| Flow mediated dilatation (mm) | 0.26  (0.07) | 0.26  (0.06) | 0.21  (0.06) | 0.20  (0.08) | 0.29  (0.06) | 0.28  (0.05) | 0.24  (0.04) | 0.24  (0.03) | -0.03  (-0.07 to 0.01) | 0.55 | 0.00  (-0.02 to 0.01) | 0.09 |
| **Variable** | **SA men with central obesity**  **(*n* = 15)** | | | | **WE men with central obesity**  **(*n* = 15)** | | | | **Pairwise comparisons SA vs. WE** | | **Pairwise comparison EX vs. CON** | |
|  | **CON**  **(fasted)** | **EX**  **(fasted)** | **CON**  **(PP)** | **EX**  **(PP)** | **CON**  **(fasted)** | **EX**  **(fasted)** | **CON**  **(PP)** | **EX**  **(PP)** | **Mean difference (95% CI)** | **Effect size (*d*)** | **Mean difference (95% CI)** | **Effect size (*d*)** |
| Baseline diameter (mm) | 4.61  (0.41) | 4.61  (0.37) | 4.61  (0.40) | 4.73  (0.43) | 4.99  (0.33) | 4.91  (0.37) | 4.99  (0.32) | 4.99  (0.36) | **-0.32**  **(-0.59 to -0.05)*** | 0.88 | 0.01  (-0.03 to 0.04) | 0.03 |
| Peak diameter (mm) | 4.77  (0.44) | 4.76  (0.40) | 4.72  (0.43) | 4.84  (0.44) | 5.20  (0.35) | 5.13  (0.39) | 5.15  (0.33) | 5.15  (0.38) | **-0.37**  **(-0.65 to -0.09)*** | 0.97 | 0.01  (-0.03 to 0.05) | 0.03 |
| **Variable** | **SA men with central obesity**  **(*n* = 15)** | | | | **WE men with central obesity**  **(*n* = 15)** | | | | **Pairwise comparisons SA vs. WE** | | **Pairwise comparison EX vs. CON** | |
|  | **CON**  **(fasted)** | **EX**  **(fasted)** | **CON**  **(PP)** | **EX**  **(PP)** | **CON**  **(fasted)** | **EX**  **(fasted)** | **CON**  **(PP)** | **EX**  **(PP)** | **Mean difference (95% CI)** | **Effect size (*d*)** | **Mean difference (95% CI)** | **Effect size (*d*)** |
| Flow mediated dilatation (%) | 3.33  (1.01) | 3.26  (0.97) | 2.42  (0.72) | 2.45  (1.00) | 4.34  (0.67) | 4.29  (0.60) | 3.28  (1.30) | 3.26  (0.75) | **-0.86**  **(-1.40 to -0.31)*** | 1.06 | -0.03  (-0.26 to 0.21) | 0.03 |
| Flow mediated dilatation (mm) | 0.16  (0.05) | 0.15  (0.05) | 0.11  (0.16) | 0.12  (0.16) | 0.22  (0.04) | 0.21  (0.03) | 0.16  (0.06) | 0.16  (0.04) | **-0.05**  **(-0.08 to -0.02)*** | 1.21 | 0.00  (-0.01 to 0.01) | 0.03 |

Data were analysed using linear mixed models with ethnicity (South Asian or white European), trial (control or exercise), and time (fasted or postprandial) included as fixed factors, and total shear rate as a covariate.

Pairwise comparisons are based on the mean absolute difference (95% CI of the mean absolute difference).

^*^Main effect of ethnicity across day one (*p*≤0.022).

A main effect of time (postprandial vs. fasted) revealed FMD% decreased in the postprandial vs. fasted state in women who were lean (-1.39 (-1.67 to -1.10)%; *d*=4.74) and men with central obesity (0.95 (-1.19 to -0.72)%; *d*=3.97). A main effect of time revealed FMDmm decreased in the postprandial vs. fasted state in women who were lean (-0.05 (-0.06 to -0.04)mm; *d*=3.59) and men with central obesity (-0.05 (-0.06 to -0.03)mm; *d=*4.49).

There were no ethnicity-by-trial interactions (*p*≥0.057) or ethnicity-by-time interactions (*p*≥0.150).

Trial-by-time interactions revealed baseline diameter was larger in the women who were lean for the postprandial vs. fasting measurement (*p*=0.047) during the exercise (0.03 (0.01 to 0.05)mm) compared to control trial (-0.01 (-0.03 to 0.02)mm). The same trial-by-time interaction was seen for the men with central obesity for the postprandial vs. fasting measurement (P=0.012) in the exercise (0.09 (0.04 to 0.14)mm) vs. control trial (0.00 (-0.04 to 0.05)mm).

There were no other trial-by-time interactions (*p*≥0.055).

CI, confidence interval; PP, postprandial; SA, South Asian; WE, white European.

**Supplementary Table 2. Day 2 endothelial function.** The baseline diameter of the brachial artery, peak diameter, absolute FMD (FMDmm), and relative FMD (FMD%) across day 2 during the control and exercise trial for South Asian and white European women who were lean, and South Asian and white European men with central obesity.

| **Variable** | **SA women who were lean**  **(*n* = 12)** | | | | **WE women who were lean**  **(*n* = 12)** | | | | **Pairwise comparisons SA vs. WE** | | **Pairwise comparison EX vs. CON** | |
| --- | --- | --- | --- | --- | --- | --- | --- | --- | --- | --- | --- | --- |
|  | **CON**  **(fasted)** | **EX**  **(fasted)** | **CON**  **(PP)** | **EX**  **(PP)** | **CON**  **(fasted)** | **EX**  **(fasted)** | **CON**  **(PP)** | **EX**  **(PP)** | **Mean difference (95% CI)** | **Effect size (*d*)** | **Mean difference (95% CI)** | **Effect size (*d*)** |
| Baseline diameter (mm) | 3.66  (0.55) | 3.68  (0.63) | 3.68  (0.62) | 3.68  (0.61) | 3.52  (0.55) | 3.54  (0.55) | 3.53  (0.56) | 3.52  (0.56) | 0.15  (-0.35 to 0.65) | 0.25 | 0.01  (-0.01 to 0.02) | 0.01 |
| Peak diameter (mm) | 3.91  (0.68) | 3.93  (0.66) | 3.85  (0.65) | 3.90  (0.66) | 3.80  (0.59) | 3.82  (0.60) | 3.75  (0.59) | 3.78  (0.61) | 0.11  (-0.42 to 0.65) | 0.18 | **0.03**  **(0.01 to 0.05)**** | 0.05 |
| **Variable** | **SA women who were lean**  **(*n* = 12)** | | | | **WE women who were lean**  **(*n* = 12)** | | | | **Pairwise comparisons SA vs. WE** | | **Pairwise comparison EX vs. CON** | |
|  | **CON**  **(fasted)** | **EX**  **(fasted)** | **CON**  **(PP)** | **EX**  **(PP)** | **CON**  **(fasted)** | **EX**  **(fasted)** | **CON**  **(PP)** | **EX**  **(PP)** | **Mean difference (95% CI)** | **Effect size (*d*)** | **Mean difference (95% CI)** | **Effect size (*d*)** |
| Flow mediated dilatation (%) | 6.89  (1.02) | 6.95  (1.07) | 4.84  (1.18) | 5.95  (1.55) | 7.99  (1.39) | 8.10  (1.49) | 6.15  (1.16) | 7.27  (1.37) | **-1.23**  **(-2.19 to -0.26)*** | 0.96 | **0.60**  **(0.32 to 0.89)**** | 0.47 |
| Flow mediated dilatation (mm) | 0.26  (0.06) | 0.25  (0.06) | 0.18  (0.05) | 0.22  (0.07) | 0.28  (0.06) | 0.29  (0.07) | 0.22  (0.04) | 0.26  (0.06) | -0.03  (-0.08 to 0.01) | 0.55 | **0.02**  **(0.01 to 0.03)**** | 0.39 |
| **Variable** | **SA men with central obesity**  **(*n* = 15)** | | | | **WE men with central obesity**  **(*n* = 15)** | | | | **Pairwise comparisons SA vs. WE** | | **Pairwise comparison EX vs. CON** | |
|  | **CON**  **(fasted)** | **EX**  **(fasted)** | **CON**  **(PP)** | **EX**  **(PP)** | **CON**  **(fasted)** | **EX**  **(fasted)** | **CON**  **(PP)** | **EX**  **(PP)** | **Mean difference (95% CI)** | **Effect size (*d*)** | **Mean difference (95% CI)** | **Effect size (*d*)** |
| Baseline diameter (mm) | 4.59  (0.38) | 4.62  (0.40) | 4.64  (0.39) | 4.63  (0.39) | 4.94  (0.33) | 4.94  (0.34) | 4.98  (0.36) | 4.96  (0.35) | **-0.32**  **(-0.59 to -0.06)*** | 0.91 | 0.00  (-0.02 to 0.02) | 0.01 |
| Peak diameter (mm) | 4.75  (0.41) | 4.77  (0.44) | 4.74  (0.41) | 4.77  (0.41) | 5.14  (0.35) | 5.15  (0.35) | 5.12  (0.37) | 5.13  (0.35) | **-0.37**  **(-0.65 to -0.09)*** | 0.98 | 0.02  (-0.00 to 0.04) | 0.05 |
| **Variable** | **SA men with central obesity**  **(*n* = 15)** | | | | **WE men with central obesity**  **(*n* = 15)** | | | | **Pairwise comparisons SA vs. WE** | | **Pairwise comparison EX vs. CON** | |
|  | **CON**  **(fasted)** | **EX**  **(fasted)** | **CON**  **(PP)** | **EX**  **(PP)** | **CON**  **(fasted)** | **EX**  **(fasted)** | **CON**  **(PP)** | **EX**  **(PP)** | **Mean difference (95% CI)** | **Effect size (*d*)** | **Mean difference (95% CI)** | **Effect size (*d*)** |
| Flow mediated dilatation (%) | 3.35  (1.00) | 3.34  (1.11) | 2.14  (1.19) | 3.10  (1.23) | 4.27  (0.77) | 4.30  (0.64) | 2.70  (0.85) | 3.60  (0.48) | **-0.74**  **(-1.35 to -0.13)*** | 0.78 | **0.47**  **(0.29 to 0.64)**** | 0.52 |
| Flow mediated dilatation (mm) | 0.16  (0.05) | 0.16  (0.06) | 0.10  (0.06) | 0.15  (0.06) | 0.21  (0.04) | 0.21  (0.03) | 0.14  (0.04) | 0.18  (0.02) | **-0.05**  **(-0.08 to -0.02)*** | 0.95 | **0.02**  **(0.01 to 0.03)**** | 0.50 |

Data were analysed using linear mixed models with ethnicity (South Asian or white European), trial (control or exercise), and time (fasted or postprandial) included as fixed factors, and total shear rate as a covariate.

Pairwise comparisons are based on the mean absolute difference (95% CI of the mean absolute difference).

^*^Main effect of ethnicity across the day two scans (*p*≤0.019).

**Main effect of trial across the day two scans (*p*≤0.001).

There was no effect of time (postprandial vs. fasted) on baseline diameter in the women who were lean (*p*=0.714, *d*=0.08) or the peak diameter in the men with central obesity (*p*=0.057, *d*=0.31).

A main effect of time revealed peak diameter was lower in the postprandial vs. fasted state in women who were lean (-0.05 (-0.07 to -0.03)mm; *d*=1.20). A main effect of time revealed baseline diameter was higher in the postprandial vs. fasted state in men with central obesity (0.03 (0.01 to 0.05)mm; *d*=1.15). A main effect of time revealed FMD% decreased for the postprandial vs. fasted measurement in women who were lean (-1.43 (-1.71 to -1.14)%; *d*=6.86) and men with central obesity (-0.93% (-1.11 to -0.75)%; *d*=3.19). A main effect of time revealed FMDmm decreased for the postprandial vs. fasted measurement in women who were lean (-0.05 (-0.06 to -0.04)mm; *d*=4.76) and men with central obesity (-0.04 (-0.05 to -0.04)mm; *d*=2.93).

There were no ethnicity-by-trial interactions (*p*≥0.420) or ethnicity-by-time interactions for the women who were lean (*p*≥0.150). There were no ethnicity-by-time interactions for the men with central obesity for baseline diameter or peak diameter (*p*≥0.434). Ethnicity-by-time interactions revealed FMD% decreased to a lesser extent for the postprandial vs. fasting measurement (*p*=0.023) in the South Asian (-0.73 (-0.98 to -0.48)%) vs. white European men with central obesity (-1.14 (-1.39 to -0.89)%).

There were no trial-by-time interactions for baseline or peak diameter (*p*≥0.203). Trial-by-time interactions revealed FMD% decreased by a lesser extent in the women who were lean in the postprandial vs. fasting measurement (*p*=0.001) for the exercise (-0.91 (-1.32 to -0.51)%) compared to control trial (-1.94 (-2.35 to -1.54)%). Trial-by-time interactions revealed FMD% decreased by a lesser extent in the men with central obesity in the postprandial vs. fasting measurement (*p*<0.001) for the exercise (-0.46 (-0.71 to -0.21)%) compared to control trial (-1.40 (-1.65 to -1.15)%). Trial-by-time interactions revealed FMDmm decreased by a lesser extent in the women who were lean for the postprandial vs. fasting measurement (*p*=0.001) for the exercise (-0.03 (-0.05 to -0.02)mm) compared to control trial (-0.07 (-0.08 to -0.05)mm). Trial-by-time interactions revealed FMDmm decreased by a lesser extent in the men with central obesity in the postprandial vs. fasting measurement (*p*<0.001) for the exercise (-0.02mm (-0.03 to -0.01)mm) compared to control trial (-0.07 (-0.08 to -0.05)mm).

CI, confidence interval; PP, postprandial; SA, South Asian; WE, white European.

**Supplementary Table 3. Postprandial metabolite and blood pressure responses.** Time averaged total area under the curve values for postprandial concentrations and resting arterial blood pressure on day 2 of the control and exercise trials after adjusting for fasting values for South Asian and white European women who were lean, and South Asian and white European men with central obesity.

| **Variable** | **SA women who were lean**  **(*n* = 12**^+^**)** | | **WE women who were lean**  **(*n* = 12**^+^**)** | | **Pairwise comparisons SA vs. WE** | | **Pairwise comparison EX vs. CON** | |
| --- | --- | --- | --- | --- | --- | --- | --- | --- |
|  | **Control** | **Exercise** | **Control** | **Exercise** | **Mean difference (95% CI)** | **Effect size (*d*)** | **Mean difference (95% CI)** | **Effect size (*d*)** |
| TAG  (mmol/L h) | 1.91  0.91 | 1.83  (0.79) | 1.53  (0.75) | 1.59  (0.52) | **0.31**  **(0.02 to 0.61)*** | 0.74 | -0.01 (-0.31 to 0.29) | 0.17 |
| Glucose (mmol/L h) | 5.02  (4.87 to 5.18) | 4.83  (4.65 to 5.00) | 4.81  (4.55 to 5.08) | 4.93  (4.64 to 5.23) | 1%  (-3% to 6%) | 0.18 | -1%  (-5% to 3%) | 0.13 |
| Insulin (pmol/L h) | 222.2  (162.5 to 303.9 | 202.5  (154.0 to 266.3) | 210.1  (171.5 to 257.3) | 172.8  (138.2 to 216.0) | 11%  (-12% to 41%) | 0.26 | -13%  (-31% to 9%) | 0.33 |
|  |  |  |  |  |  |  |  |  |

| **Variable** | **SA women who were lean**  **(*n* = 12**^+^**)** | | **WE women who were lean**  **(*n* = 12**^+^**)** | | **Pairwise comparisons SA vs. WE** | | **Pairwise comparison EX vs. CON** | |
| --- | --- | --- | --- | --- | --- | --- | --- | --- |
|  | **Control** | **Exercise** | **Control** | **Exercise** | **Mean difference (95% CI)** | **Effect size (*d*)** | **Mean difference (95% CI)** | **Effect size (*d*)** |
| NEFA (mmol/L h) | 0.38  (0.35 to 0.42) | 0.41  (0.35 to 0.50) | 0.34  (0.31 to 0.38) | 0.28  (0.26 to 0.31) | **29%**  **(15% to 44%)*** | 1.41 | -5%  (-15% to 6%) | 0.23 |
| PRDX4 (ng/mL h) | 7.33  (1.94) | 7.27  (1.34) | 9.05  (3.47) | 7.11  (1.79) | -0.79  (-1.79 to 0.21) | 0.98 | **-1.00**  **(-1.92 to -0.09)**** | 0.41 |
| SOD3 (ng/mL h) | 5.80  (3.70) | 5.50  (2.19) | 5.37  (2.75) | 4.27  (2.17) | **0.83**  **(0.08 to 1.57)*** | 0.51 | -0.70  (-1.44 to 0.04) | 0.32 |
| SBP  (mmHg h) | 102  (99 to 106) | 104  (99 to 110) | 109  (106 to 112) | 106  (105 to 108) | **-4%**  **(-7% to -1%)*** | 0.57 | 0%  (-3% to 3%) | 0.01 |

| **Variable** | **SA women who were lean**  **(*n* = 12**^+^**)** | | **WE women who were lean**  **(*n* = 12**^+^**)** | | **Pairwise comparisons SA vs. WE** | | **Pairwise comparison EX vs. CON** | |
| --- | --- | --- | --- | --- | --- | --- | --- | --- |
|  | **Control** | **Exercise** | **Control** | **Exercise** | **Mean difference (95% CI)** | **Effect size (*d*)** | **Mean difference (95% CI)** | **Effect size (*d*)** |
| DBP  (mmHg h) | 72  (70 to 75) | 71  (68 to 74) | 71  (69 to 74) | 70  (68 to 72) | 1%  (-2% to 5%) | 0.15 | -2%  (-5% to 2%) | 0.18 |
| **Variable** | **SA men with central obesity**  **(*n* = 15)** | | **WE men with central obesity**  **(*n* = 15)** | | **Pairwise comparisons SA vs. WE** | | **Pairwise comparison EX vs. CON** | |
|  | **Control** | **Exercise** | **Control** | **Exercise** | **Mean difference (95% CI)** | **Effect size (*d*)** | **Mean difference (95% CI)** | **Effect size (*d*)** |
| TAG (mmol/L h) | 3.67  (1.04) | 3.13  (0.88) | 3.23  (1.24) | 2.96  (1.13) | 0.55  (0.20 to 0.90) | 0.65 | -0.25  (-0.60 to 0.10) | 0.39 |
| Glucose (mmol/L h) | 6.05  (5.69 to 6.43) | 6.06  (5.85 to 6.27) | 6.29  (5.83 to 6.79) | 6.32  (5.94 to 6.72) | -4%  (-9% to 2%) | 0.27 | 0%  (-5% to 6%) | 0.02 |

| **Variable** | **SA men with central obesity**  **(*n* = 15)** | | **WE men with central obesity**  **(*n* = 15)** | | **Pairwise comparisons SA vs. WE** | | **Pairwise comparison EX vs. CON** | |
| --- | --- | --- | --- | --- | --- | --- | --- | --- |
|  | **Control** | **Exercise** | **Control** | **Exercise** | **Mean difference (95% CI)** | **Effect size (*d*)** | **Mean difference (95% CI)** | **Effect size (*d*)** |
| Insulin (pmol/L h) | 376.3  (318.4 to 444.6) | 343.5  (312.3 to 377.8) | 341.9  (299.0 to 390.8) | 379.6  (325.4 to 442.8) | 0%  (-12% to 14%) | 0.00 | 1%  (-12% to 15%) | 0.01 |
| NEFA (mmol/L h) | 0.36  (0.04) | 0.38  (0.03) | 0.35  (0.04) | 0.40  (0.04) | -0.01  (-0.04 to 0.03) | 0.40 | **0.04**  **(0.01 to 0.07)**** | 0.86 |
| PRDX4 (ng/mL h) | 12.35  (10.75 to 14.18) | 11.35  (10.12 to 12.72) | 12.25  (10.14 to 14.79) | 11.20  (9.52 to 13.18) | 1%  (-12% to 17%) | 0.03 | -8%  (-21% to 6%) | 0.22 |
| SOD3 (ng/mL h) | 5.10  (1.97) | 5.60  (1.22) | 6.02  (1.50) | 5.75  (1.72) | -0.54  (-1.13 to 0.06) | 0.23 | 0.11  (-0.48 to 0.71) | 0.02 |

| **Variable** | **SA men with central obesity**  **(*n* = 15)** | | **WE men with central obesity**  **(*n* = 15)** | | **Pairwise comparisons SA vs. WE** | | **Pairwise comparison EX vs. CON** | |
| --- | --- | --- | --- | --- | --- | --- | --- | --- |
|  | **Control** | **Exercise** | **Control** | **Exercise** | **Mean difference (95% CI)** | **Effect size (*d*)** | **Mean difference (95% CI)** | **Effect size (*d*)** |
| SBP  (mmHg h) | 129  (10) | 126  (9) | 130  (11) | 126  (9) | -1  (-3 to 2) | 0.84 | **-3**  **(-5 to -1)**** | 0.55 |
| DBP  (mmHg h) | 81  (8) | 78  (6) | 79  (8) | 79  (6) | 1  (-1 to 2) | 0.50 | -1  (-3 to 0) | 0.66 |

Data were analysed using linear mixed models with ethnicity (South Asian or White European), trial (control or exercise), and ethnicity-by-trial interaction included as fixed factors. Within groups, normally distributed data are presented as mean (SD), and non-normally distributed data are presented as geometric mean (95% confidence interval).

For normally distributed data, pairwise comparisons are based on the mean absolute difference (95% CI of the mean absolute difference). For non-normally distributed data, comparisons are based on the ratio of the geometric mean (95% CI for the ratio of geometric means).

^*^Main effect of ethnicity (*p*<0.040). **Main effect of trial (*p*<0.033). ^+^In both women groups who were lean, blood pressure responses have an *n* of 12. Plasma metabolites have an *n* of 10.

CI, confidence interval; DBP, diastolic blood pressure; NEFA, non-esterified fatty acids; PRDX4, peroxiredoxin 4; SOD3, superoxide dismutase 3; SA, South Asian; SBP, systolic blood pressure; TAG, triacylglycerol; WE, white European.

**Supplementary Table 4.** Fasting plasma metabolite concentrations and baseline relative flow-mediated dilatation

| **Variable** | **Women who were lean** | | | | **Men with central obesity** | | | |
| --- | --- | --- | --- | --- | --- | --- | --- | --- |
|  | **SA**  **(n = 12)** | **WE**  **(n = 12)** | **Pairwise comparisons**  **SA vs. WE women** | | **SA**  **(n = 15)** | **WE**  **(n = 15)** | **Pairwise comparisons**  **SA vs. WE men** | |
|  |  |  | **Mean difference**  **(95% CI)** | **Effect size (*d*)** |  |  | **Mean difference (95% CI)** | **Effect size (*d*)** |
| TC  (mmol/L) | 4.44  (0.86) | 4.53  (0.53) | -0.09  (-0.56 to 0.39) | 0.15 | 6.00  (1.30) | 5.35  (1.23) | 0.65  (-0.25 to 1.55) | 0.54 |
| HDL  (mmol/L) | 1.28  (1.12 to 1.46) | 1.66  (1.45 to 1.91) | **-23%**  **(-36% to -7%)^*^** | 1.07 | 1.12  (0.21) | 1.13  (0.21) | -0.01  (-0.16 to 0.14) | 0.06 |
| LDL  (mmol/L) | 2.46  (2.10 to 2.88) | 2.03  (1.72 to 2.39) | 21%  (-3% to 52%) | 0.54 | 3.44  (0.93) | 2.94  (0.81) | 0.50  (-0.13 to 1.13) | 0.59 |

| **Variable** | **Women who were lean** | | | | **Men with central obesity** | | | |
| --- | --- | --- | --- | --- | --- | --- | --- | --- |
|  | **SA**  **(n = 12)** | **WE**  **(n = 12)** | **Pairwise comparisons**  **SA vs. WE women** | | **SA**  **(n = 15)** | **WE**  **(n = 15)** | **Pairwise comparisons**  **SA vs. WE men** | |
|  |  |  | **Mean difference**  **(95% CI)** | **Effect size (*d*)** |  |  | **Mean difference (95% CI)** | **Effect size (*d*)** |
| TAG  (mmol/L) | 1.09  (0.57) | 1.11  (0.50) | -0.02  (-0.30 to 0.26) | 0.09 | 1.47  (0.43) | 1.73  (0.71) | -0.26  (-0.58 to 0.05) | 0.30 |
| Glucose (mmol/L) | 4.57  (4.39 to 4.76) | 4.38  (4.20 to 4.57) | 4%  (-2% to 11%) | 0.08 | 5.42  (5.09 to 5.76) | 5.62  (5.28 to 5.97) | -4%  (-12% to 5%) | 0.30 |
| Insulin  (pmol/L) | 27.6  (18.8 to 40.4) | 19.4  (13.0 to 29.0) | 42%  (-18% to 148%) | 0.28 | 54.4  (38.0 to 77.9) | 52.3  (36.5 to 75.0) | 4%  (-37% to 73%) | 0.06 |

| **Variable** | **Women who were lean** | | | | **Men with central obesity** | | | |
| --- | --- | --- | --- | --- | --- | --- | --- | --- |
|  | **SA**  **(n = 12)** | **WE**  **(n = 12)** | **Pairwise comparisons**  **SA vs. WE women** | | **SA**  **(n = 15)** | **WE**  **(n = 15)** | **Pairwise comparisons**  **SA vs. WE men** | |
|  |  |  | **Mean difference**  **(95% CI)** | **Effect size (*d*)** |  |  | **Mean difference (95% CI)** | **Effect size (*d*)** |
| NEFA  (mmol/L) | 0.39  (0.32 to 0.46) | 0.42  (0.35 to 0.51) | -9%  (-29% to 18%) | 0.21 | 0.45  (0.40 to 0.50) | 0.60  (0.53 to 0.67) | **-25%**  **(-37% to -11%)^*^** | 0.73 |
| CRP  (mg/L) | 0.79  (0.39 to 1.60) | 0.72  (0.34 to 1.50) | 10%  (-60% to 206%) | 0.08 | 2.00  (1.14 to 3.50) | 1.06  (0.61 to 1.86) | 88%  (-15% to 315%) | 0.59 |
| TNF-alpha (pg/mL) | 1.12  (0.97 to 1.30) | 0.88  (0.75 to 1.03) | **28%**  **(3% to 58%)^*^** | 0.89 | 1.37  (0.35) | 1.22  (0.46) | 0.15  (-0.14 to 0.44) | 0.38 |

| **Variable** | **Women who were lean** | | | | **Men with central obesity** | | | |
| --- | --- | --- | --- | --- | --- | --- | --- | --- |
|  | **SA**  **(n = 12)** | **WE**  **(n = 12)** | **Pairwise comparisons**  **SA vs. WE women** | | **SA**  **(n = 15)** | **WE**  **(n = 15)** | **Pairwise comparisons**  **SA vs. WE men** | |
|  |  |  | **Mean difference**  **(95% CI)** | **Effect size (*d*)** |  |  | **Mean difference (95% CI)** | **Effect size (*d*)** |
| IL-6  (pg/mL) | 1.44  (0.65) | 1.16  (0.62) | 0.28  (-0.32 to 0.87) | 0.39 | 2.81  (1.77 to 4.45) | 1.75  (1.10 to 2.77) | 60%  (-16% to 208%) | 0.55 |
| Peroxiredoxin-4 (ng/mL) | 6.01  (1.99) | 7.75  (2.37) | -1.73  (-3.74 to 0.27) | 0.71 | 7.88  (6.31 to 9.85) | 8.86  (7.09 to 11.07) | -11%  (-35% to 22%) | 0.26 |
| Superoxide dismutase-3 (ng/mL) | 5.32  (2.92) | 4.63  (2.77) | 0.69  (-1.76 to 3.14) | 0.25 | 5.38  (2.39) | 5.87  (2.12) | -0.49  (-1.92 to 0.94) | 0.57 |

| **Variable** | **Women who were lean** | | | | **Men with central obesity** | | | |
| --- | --- | --- | --- | --- | --- | --- | --- | --- |
|  | **SA**  **(n = 12)** | **WE**  **(n = 12)** | **Pairwise comparisons**  **SA vs. WE women** | | **SA**  **(n = 15)** | **WE**  **(n = 15)** | **Pairwise comparisons**  **SA vs. WE men** | |
|  |  |  | **Mean difference**  **(95% CI)** | **Effect size (*d*)** |  |  | **Mean difference (95% CI)** | **Effect size (*d*)** |
| Progesterone  (ng/mL) | 0.97  (0.79 to 1.14) | 0.93  (0.75 to 1.11) | 0.04  (-0.21 to 0.29) | 0.12 | N/A | N/A | N/A | N/A |
| 17 β estradiol  (pg/mL) | 53.6  (10.2) | 52.6  (9.8) | 1.0  (-9.7 to 11.7) | 0.22 | N/A | N/A | N/A | N/A |
| Baseline FMD (%) | 6.93  (1.19) | 8.14  (1.36) | **-1.21**  **(-2.29 to -0.13)^*^** | 0.94 | 3.33  (1.01) | 4.34  (0.67) | **-1.01**  **(-1.58 to -0.44)^*^** | 1.09 |

Within groups, normally distributed data are mean (SD) and non-normally distributed data are geometric mean (95% CI).

For normally distributed data, ethnic comparisons are based on the mean absolute difference (95% CI of the mean absolute difference between ethnicities). For non-normally distributed data, ethnic comparisons are based on the ratio of the geometric means (95% CI for the ratio of geometric means between ethnicities).

CI, confidence interval; CRP, C-reactive protein; DBP, diastolic blood pressure; HDL, high-density lipoprotein cholesterol; FMD, flow-mediated dilatation; IL-6, interleukin 6; LDL, low-density lipoprotein cholesterol; NEFA, non-esterified fatty acids; SA, South Asian; SBP, systolic blood pressure; TAG, triacylglycerol; TC, total cholesterol; TNF-alpha, tumour necrosis factor alpha; WE, white European.

**^*^**Main effect of ethnicity (*p*≤0.045).

**Supplementary Table 5. Exercise responses.** Responses to treadmill walking in South Asian and white European women who were lean, and South Asian and white European men with central obesity**.**

| **Variable** | **Women who were lean** | | | | **Men with central obesity** | | | |
| --- | --- | --- | --- | --- | --- | --- | --- | --- |
|  | **SA**  **(n = 12)** | **WE**  **(n = 12)** | **Pairwise comparisons**  **SA vs. WE women** | | **SA**  **(n = 15)** | **WE**  **(n = 15)** | **Pairwise comparisons**  **SA vs. WE men** | |
|  |  |  | **Mean difference**  **(95% CI)** | **Effect size (*d*)** |  |  | **Mean difference (95% CI)** | **Effect size (*d*)** |
| Treadmill gradient (%) | 3.3 (1.7) | 3.7 (1.4) | -0.4 (-1.8 to 0.9) | 0.28 | 1.9 (1.7) | 2.8 (1.6) | -0.8 (-2.1 to 0.3) | 0.51 |
| Heart rate (beats/min) | 149 (14) | 148 (14) | 1 (-10 to 13) | 0.12 | 136 (12) | 132 (12) | 4 (-5 to 13) | 0.33 |
| Rating of perceived exertion | 13 (1) | 14 (1) | 0 (-1 to 1) | 0.23 | 11 (1) | 11 (2) | 0 (-1 to 1) | 0.19 |
| Absolute oxygen uptake (L/min) | 1.30 (0.19) | 1.53 (0.26) | **-0.23 (-0.43 to -0.04)^*^** | 1.04 | 1.61 (0.43) | 2.08 (0.45) | **-0.47 (-0.81 to -0.15)^*^** | 1.08 |
| **Variable** | **Women who were lean** | | | | **Men with central obesity** | | | |
|  | **SA**  **(n = 12)** | **WE**  **(n = 12)** | **Pairwise comparisons**  **SA vs. WE women** | | **SA**  **(n = 15)** | **WE**  **(n = 15)** | **Pairwise comparisons**  **SA vs. WE men** | |
|  |  |  | **Mean difference (95% CI)** | **Effect size (*d*)** |  |  | **Mean difference (95% CI)** | **Effect size**  **(*d*)** |
| Relative oxygen uptake (mL/kg/min) | 20.9 (2.4) | 23.7 (4.7) | -2.8 (-5.9 to 0.4) | 0.74 | 26.1 (5.0) | 29.2 (4.4) | -3.1 (-6.6 to 0.4) | 0.66 |
| Percent V̇O_2_ peak (%) | 60.4 (3.7) | 58.6 (1.6) | 1.8 (-0.6 to 4.2) | 0.62 | 60.3 (3.2) | 58.9 (2.8) | 1.5 (-0.7 to 3.7) | 0.50 |
| Respiratory exchange ratio | 0.80 (0.05) | 0.83 (0.04) | -0.03 (-0.08 to 0.01) | 0.67 | 0.87 (0.05) | 0.87 (0.04) | 0.00 (-0.03 to 0.03) | 0.03 |
| Fat oxidation (g) | 26.4 (8.4) | 26.2 (7.7) | 0.2 (-6.6 to 7.0) | 0.03 | 21.4 (8.1) | 28.9 (14.3) | -7.5 (-16.1 to 1.2) | 0.64 |
| Carbohydrate oxidation (g) | 32.2 (20.4) | 51.7 (19.7) | **-19.5 (-36.5 to -2.6)^*^** | 0.97 | 70.9 (31.2) | 88.9 (21.8) | -18.0 (-38.1 to 2.1) | 0.67 |

| **Variable** | **Women who were lean** | | | | **Men with central obesity** | | | |
| --- | --- | --- | --- | --- | --- | --- | --- | --- |
|  | **SA**  **(n = 12)** | **WE**  **(n = 12)** | **Pairwise comparisons**  **SA vs. WE women** | | **SA**  **(n = 15)** | **WE**  **(n = 15)** | **Pairwise comparisons**  **SA vs. WE men** | |
|  |  |  | **Mean difference (95% CI)** | **Effect size (*d*)** |  |  | **Mean difference (95% CI)** | **Effect size**  **(*d*)** |
| Total energy expenditure (kJ) | 1545 (225) | 1849 (317) | **-304 (-537 to -71)^*^** | 1.10 | 1971 (538) | 2549 (528) | **-579 (-977 to -180)^*^** | 1.09 |

Data were analysed using linear mixed models with ethnicity (South Asian or white European) included as a fixed factor.

Comparisons are based on the mean absolute difference (95% confidence interval of the mean absolute difference).

**^*^**Main effect of ethnicity (*p*<0.026).

CI, confidence interval; SA, South Asian; WE, white European.

**
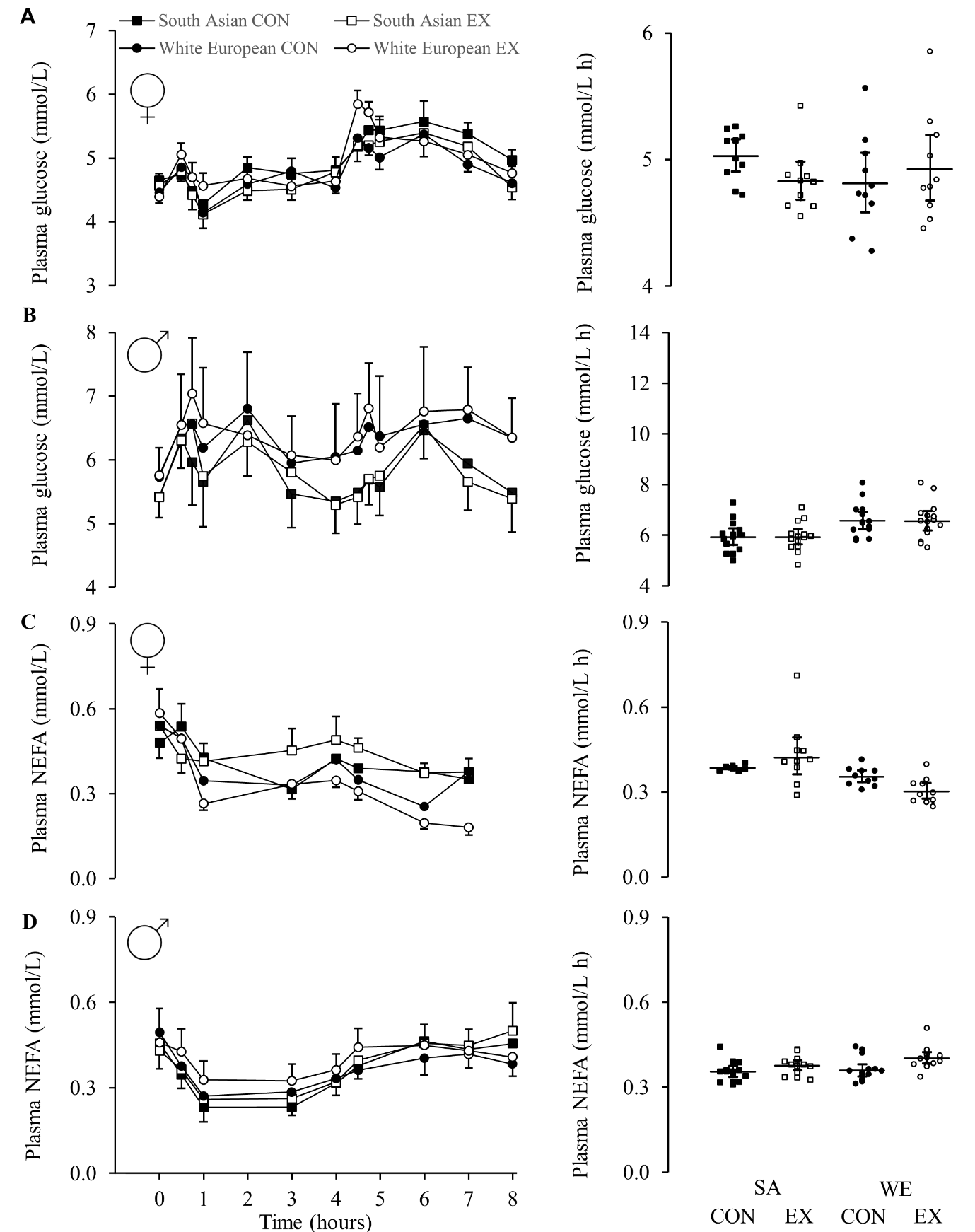
**

**Supplementary Figure 1.** **Individual glucose and NEFA responses.** Glucose (panels A and B) and NEFA (panels C and D) responses on day 2 of the control and exercise trials. Panels A and C are for the women who are lean (*n* = 10 in both groups), panels B and D are for the men with central obesity (*n* = 15 in both groups). Data presented as the geometric mean (95% CI). Panels on right-side display individual time averaged total area under the curve (TAUC) values after adjustment for fasting concentrations. Responses were assessed using linear mixed models with ethnicity, trial, and ethnicity-by-trial interaction modelled as fixed factors, and fasting concentrations as a covariate. Breakfast fed at 0 hours; lunch fed at 4 hours. CI, confidence interval; CON, control; EX, exercise; NEFA; non-esterified fatty acids; SA, South Asian; WE, white European.


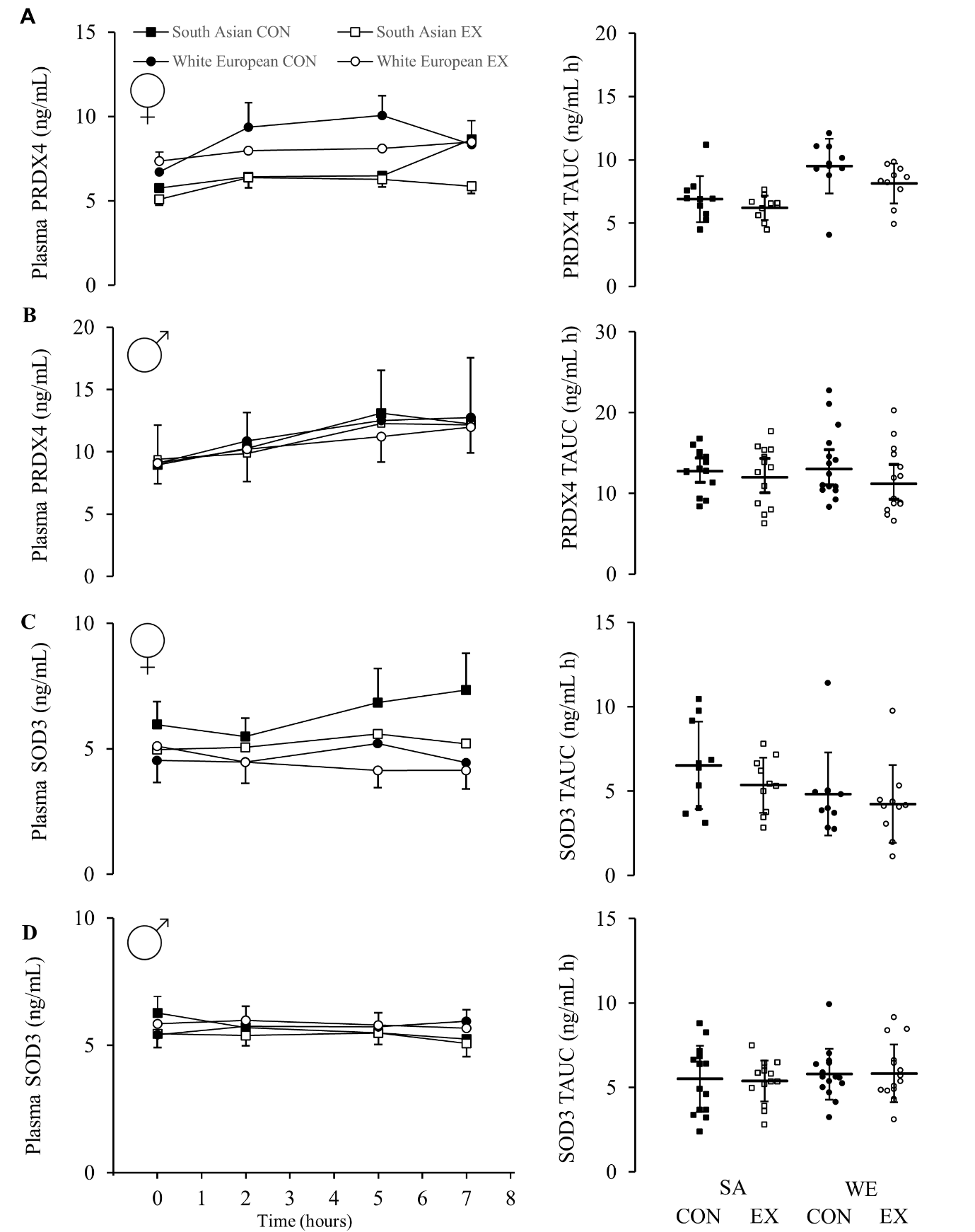


**Supplementary Figure 2.** **Individual PRDX4 and SOD3 responses.** Peroxiredoxin-4 (PRDX4) (panels A and B) and superoxide dismutase 3 (SOD3) (panels C and D) responses on day 2 of the control and exercise trials. Panels A and C are for the women who were lean (*n* = 10 in both groups), panels B and D are for the men with central obesity (*n* = 15 in both groups). PRDX4 presented as the mean (SEM) for the women and geometric mean (95% CI) for men. SOD3 presented as the mean (SEM). Panels on the right-side display time averaged total area under the curve (TAUC) values after adjustment for differences in fasting concentrations. Responses were assessed using linear mixed models with ethnicity, trial, and ethnicity-by-trial interaction modelled as fixed factors, and fasting concentrations as a covariate. Breakfast fed at 0 hours; lunch fed at 4 hours. CI, confidence interval; CON, control; EX, exercise; PRDX4, peroxiredoxin 4; SA, South Asian; SEM, standard error of the mean; SOD3, superoxide dismutase 3; WE, white European.


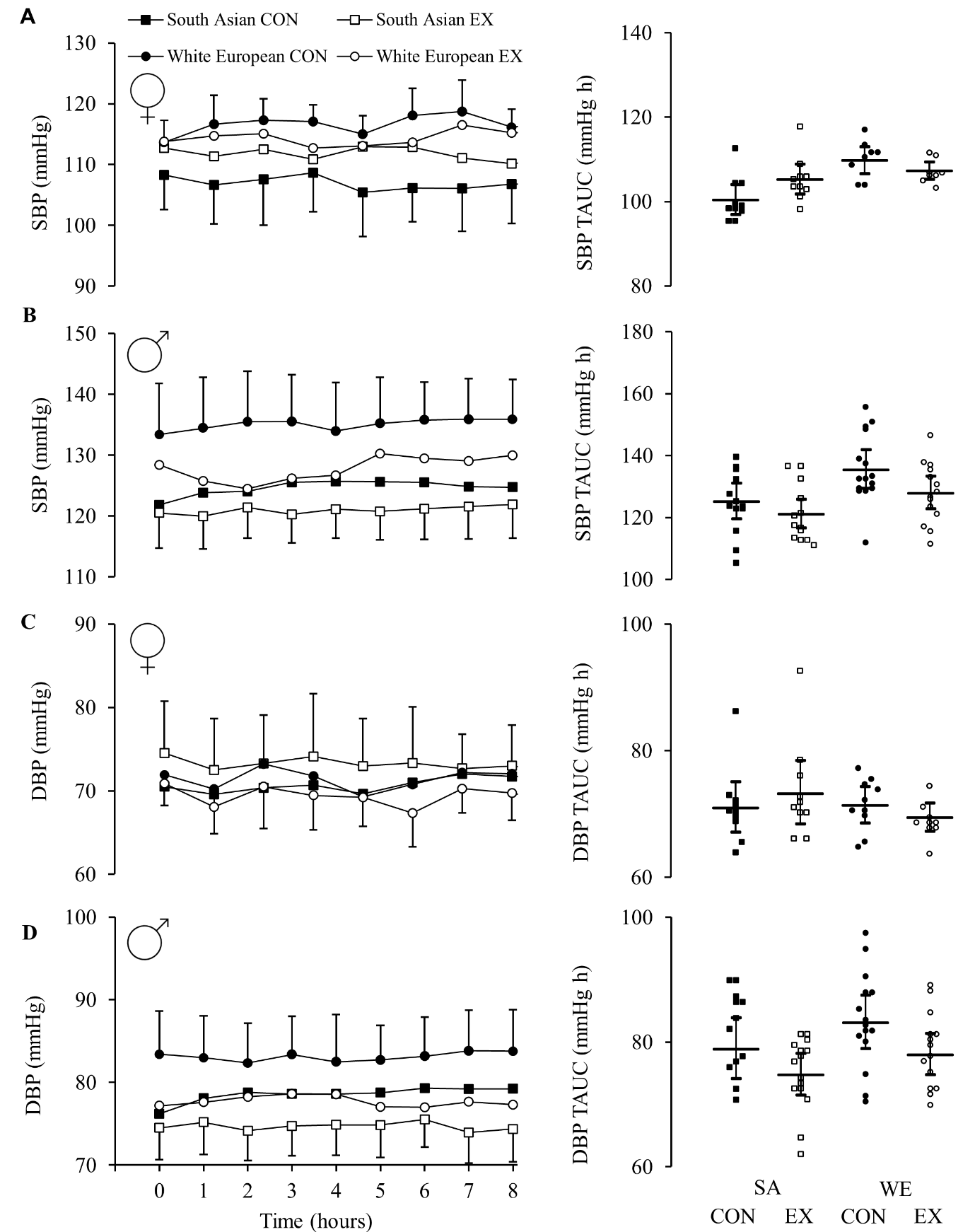


**Supplementary Figure 3. Individual SBP and DBP responses.** Day two SBP (panels A and B) and DBP (panels C and D) in the control and exercise trials. Panels A and C are for the women who were lean (*n* = 12 in both groups), panels B and D are for the men with central obesity (*n* = 15 in both groups). Data presented as geometric mean (95% CI) for women and mean (SEM) for men. Panels on right-side display individual time averaged total area under the curve (TAUC) values after adjustment for differences in fasting measurements. Responses were assessed using linear mixed models with ethnicity, trial, and ethnicity-by-trial interaction modelled as fixed factors, and fasting concentrations as a covariate. Breakfast fed at 0 hours; lunch fed at 4 hours. CI, confidence interval; CON, control; DBP, diastolic blood pressure; EX, exercise; SA, South Asian; SBP, systolic blood pressure; SEM, standard error of the mean; WE, White European.
